# CHD7 and 53BP1 regulate distinct pathways for the re-ligation of DNA double-strand breaks

**DOI:** 10.1038/s41467-020-19502-5

**Published:** 2020-11-13

**Authors:** Magdalena B. Rother, Stefania Pellegrino, Rebecca Smith, Marco Gatti, Cornelia Meisenberg, Wouter W. Wiegant, Martijn S. Luijsterburg, Ralph Imhof, Jessica A. Downs, Alfred C. O. Vertegaal, Sébastien Huet, Matthias Altmeyer, Haico van Attikum

**Affiliations:** 1 Department of Human Genetics, Leiden University Medical Center, Leiden, The Netherlands; 2 Department of Molecular Mechanisms of Disease, University of Zurich, Zurich, Switzerland; 3 Univ Rennes, CNRS, IGDR (Institut de génétique et développement de Rennes)—UMR 6290, BIOSIT—UMS3480, F-35000 Rennes, France; 4 The Institute of Cancer Research, Royal Cancer Hospital, London, UK; 5 Department of Cell and Chemical Biology, Leiden University Medical Center, Leiden, The Netherlands; 6 Institut Universitaire de France, Paris, France

**Keywords:** Acetylation, Chromatin remodelling, DNA damage response, Double-strand DNA breaks, Non-homologous-end joining

## Abstract

Chromatin structure is dynamically reorganized at multiple levels in response to DNA double-strand breaks (DSBs). Yet, how the different steps of chromatin reorganization are coordinated in space and time to differentially regulate DNA repair pathways is insufficiently understood. Here, we identify the Chromodomain Helicase DNA Binding Protein 7 (CHD7), which is frequently mutated in CHARGE syndrome, as an integral component of the non-homologous end-joining (NHEJ) DSB repair pathway. Upon recruitment via PARP1-triggered chromatin remodeling, CHD7 stimulates further chromatin relaxation around DNA break sites and brings in HDAC1/2 for localized chromatin de-acetylation. This counteracts the CHD7-induced chromatin expansion, thereby ensuring temporally and spatially controlled ‘chromatin breathing’ upon DNA damage, which we demonstrate fosters efficient and accurate DSB repair by controlling Ku and LIG4/XRCC4 activities. Loss of CHD7-HDAC1/2-dependent cNHEJ reinforces 53BP1 assembly at the damaged chromatin and shifts DSB repair to mutagenic NHEJ, revealing a backup function of 53BP1 when cNHEJ fails.

## Abstract (web summary)

Chromatin is dynamically remodeled in response to DNA damage in favour of repair. Here the authors reveal how the chromatin remodeler CHD7 and chromatin binding protein 53BP1 regulate distinct DNA repair pathways.

## Introduction

DNA double-strand breaks (DSBs) are one of the most challenging forms of DNA damage that jeopardize genome integrity. If left unrepaired or repaired inaccurately, they can lead to chromosomal rearrangements or loss of genetic information, thereby triggering cell death and contributing to human diseases, including cancer^1^. To prevent genetic instability and associated diseases, cells have evolved mechanisms for the signaling and repair of DSBs, collectively referred to as the DNA damage response^1^. DSBs can be repaired by either homologous recombination (HR) or non-homologous end-joining (NHEJ). HR is mostly active in the S and G2 phases of the cell cycle and requires end-resection to form large stretches of single-stranded DNA (ssDNA) at DSBs. The ssDNA becomes bound by RPA, which is subsequently replaced by RAD51 in a manner dependent on BRCA1, PALB2, and BRCA2. This facilitates error-free repair of DSBs by using the undamaged sister chromatid as a template^2^. In contrast, canonical NHEJ (cNHEJ) is initiated by the binding of the Ku70/Ku80 (Ku) heterodimer to the DSB ends, followed by activation of DNA-PKcs and recruitment of the XLF–XRCC4–LIG4 complex, which, stimulated by XRCC4 and XLF paralogue PAXX, seals the broken ends^3^. An alternative NHEJ (altNHEJ) pathway also exists, which relies on the XRCC1–DNA ligase III complex and joins DSB ends in an error-prone manner using microhomology^3^.

The choice between HR and NHEJ throughout the cell cycle is regulated at multiple levels, including activation of the ATM kinase, which is recruited to DSBs by the MRE11–RAD50–NBS1 (MRN) complex. MRN and ATM initiate a signaling cascades driven by the ATM-dependent phosphorylation of the histone variant H2A.X around DSBs, forming γH2AX, which serves as a platform for MDC1 loading^4^. ATM-dependent phosphorylation of MDC1 recruits the E3 ubiquitin ligase RNF8, which ubiquitylates histone H1, thereby promoting the accrual of another E3 ubiquitin ligase RNF168. RNF168 further decorates DSB-flanking chromatin with ubiquitin conjugates to promote the assembly of 53BP1 and the BRCA1–Abraxas–RAP80–MERIT40 (BRCA1-A) complex and regulate DSB repair^4^. 53BP1 limits DNA end-resection through recruitment of various effector proteins such as RIF1 and the Shieldin complex^5,6^, whereas the BRCA1-A complex suppresses HR by sequestering BRCA1 away from the repair site^7,8^.

53BP1 is recruited to DSBs when histone H2A becomes ubiquitylated at lysine 15 (H2AK15) through the consecutive activities of RNF8 and RNF168^9^. The recognition of this histone mark by 53BP1 involves a peptide segment termed the ubiquitin-dependent recruitment motif, but stable binding of 53BP1 requires its simultaneous association with dimethylated lysine 20 in histone H4 (H4K20me2) through its tandem tudor domain^9–11^, making it a bivalent histone mark reader^12^. High levels of H4K20me2 in unreplicated chromatin promote 53BP1 recruitment, whereas replication-coupled dilution of H4K20me2 during S-phase progression lowers 53BP1 recruitment at replicated areas of the genome and promotes HR factor recruitment^13–15^. Besides the histone marks that recruit 53BP1 to DSBs, several other histone modifications and structural chromatin changes driven by ATP-dependent chromatin remodelers affect the DSB response^16^. However, the full repertoire of chromatin changes that orchestrate DSB repair and impact 53BP1 function remains to be explored.

Here, we performed a 53BP1 gain-of-function RNAi screen with the aim to identify chromatin modifiers that affect the DSB response. We identified the CHARGE syndrome protein Chromodomain Helicase DNA-Binding Protein 7 (CHD7), whose deficiency causes excessive assembly of 53BP1 at chromatin surrounding DSBs. We show that CHD7, along with its binding partners histone de-acetylase 1 and 2 (HDAC1/2), accumulates early at DSB-flanking chromatin regions devoid of 53BP1. The sequential activities of CHD7 and HDAC1/2 ensure rapid expansion followed by re-compaction of the damaged chromatin, respectively. This ensures proper binding and controlled retention of NHEJ factors at DNA breaks. Consequently, CHD7 promotes faithful cNHEJ without or only minimal mutagenic DNA end processing, which is distinct from more erroneous NHEJ after limited, 53BP1-constrained end resection.

## Results

### CHD7 curtails RNF8/RNF168-dependent recruitment of 53BP1

**Fig. 1:**
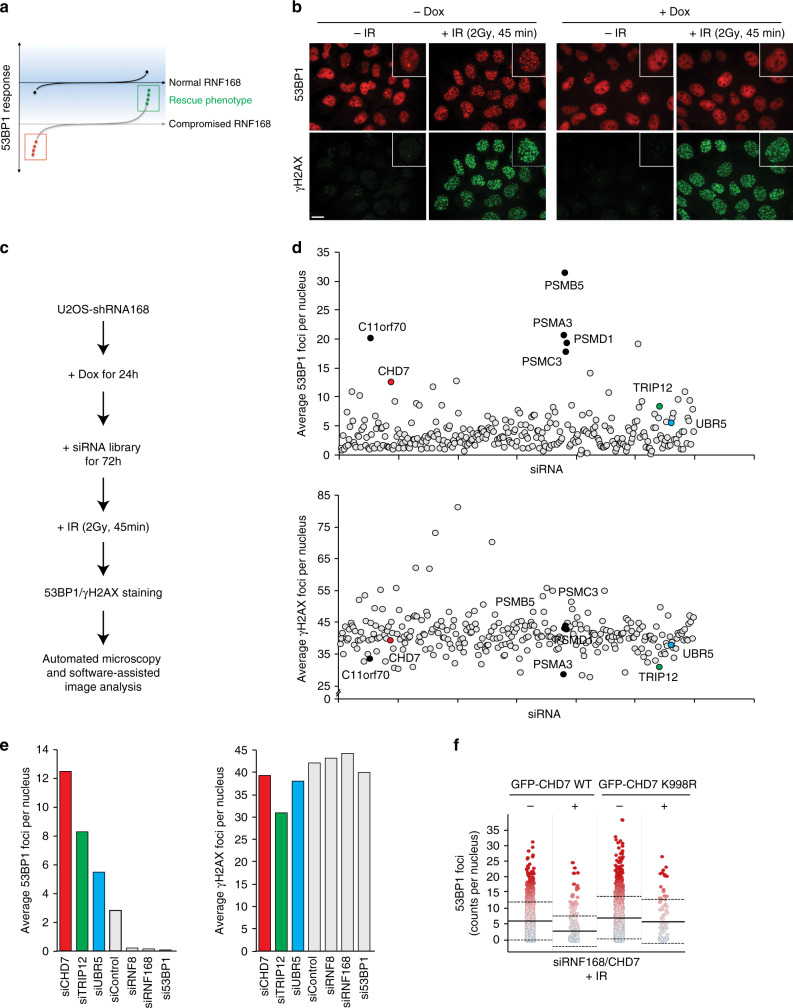
CHD7 curtails RNF8/RNF168-dependent recruitment of 53BP1. **a** Schematic representation of the 53BP1 gain-of-function screen to reveal rescue phenotypes from experimentally evoked compromised recruitment of 53BP1 to sites of DNA damage. **b** Representative immunofluorescent images from the screen showing lack of 53BP1 nuclear body and foci formation in U2OS-shRNF168 cells upon induction of the shRNA. **c** Experimental design of the 53BP1 gain-of-function RNAi screen. **d**, **e** The average number of 53BP1 and γH2AX foci per nucleus upon the indicated siRNA-mediated depletions in the U2OS-shRNF168 background. **f** Quantification of single-cell QIBC analysis of >1000 cells per plasmid transfection. U2OS cells were treated with the indicated siRNAs and transfected with the indicated siRNA-resistant GFP–CHD7 fusions. Cells were exposed to 0.5 Gy of IR and 53BP1 foci were quantified after 15 min in control cells (GFP-negative) and CHD7-transfected (GFP-positive) cells. Mean (solid line) and standard deviation from the mean (dashed lines) are indicated. Scale bar 10 µm. Source data are provided as a Source Data file.

To uncover new chromatin modifiers that might act upstream of the DSB repair pathway choice modulator 53BP1 during the DSB response, we performed a targeted 53BP1 rescue or gain-of-function RNAi screen (Fig. 1a). Stable U2OS cells expressing an inducible shRNA against the ubiquitin E3 ligase RNF168^17^ were employed to interrogate 53BP1 recruitment under conditions of dampened RNF168 expression. As expected, doxycycline-induced depletion of RNF168 greatly reduced the formation of 53BP1 nuclear bodies and ionizing radiation (IR)-induced foci without negatively affecting the upstream γH2AX signal (Fig. 1b). We used these conditions in conjunction with a custom-designed siRNA library comprising a set of nuclear and chromatin-associated proteins to screen for regained 53BP1 accumulation at IR-induced DSBs (Fig. 1c and Supplementary Data 1). Reassuringly, and consistent with our previous work^17^, several proteasome subunits scored in this screen (e.g., PSMB5, PSMA3, PSMD1, and PSMC3). Moreover, the two ubiquitin E3 ligases UBR5 and TRIP12, which control proteasomal RNF168 degradation and thereby the extent of IR-induced chromatin ubiquitylation^17^, also scored. Interestingly, we identified that co-depletion of the chromatin remodeler CHD7 also strongly restored 53BP1 foci formation (4-fold increase over the screen average, Supplementary Data 1) without leading to elevated levels of γH2AX foci (Fig. 1d, e). CHD7 is frequently mutated in the severe autosomal dominant congenital genetic disorder CHARGE, for coloboma, heart defect, atresia choanae, restricted growth and development, genital abnormality, and ear abnormality^18^, and has not yet been linked to the 53BP1-dependent response to DNA damage. CHARGE patients have heterozygous mutations in CHD7, which are mostly truncating mutations. Missense mutations occur in a minority of patients and partial or full deletions of the CHD7 gene are rare^19^.

Individual validation experiments confirmed that CHD7 loss does not impact 53BP1, RNF168, or RNF8 expression, but restores 53BP1 foci formation in RNF168-depleted cells, as well as in RNF8-depleted cells (Supplementary Data 2 and Supplementary Fig. 1a–c). Re-expression of siRNA-resistant green fluorescent protein (GFP)-tagged wildtype (WT), but not ATPase-dead (K998R) CHD7^20^ largely suppressed 53BP1 foci formation in these cells (Fig. 1f). Besides 53BP1 foci formation, IR-induced ubiquitin chain formation was also partially restored, suggesting that RNF8/RNF168-driven chromatin ubiquitylation is hyperactive in CHD7-depleted cells (Supplementary Fig. 1d). Consistently, when we assessed 53BP1 foci formation upon CHD7 loss in RNF8/RNF168-proficient cells, we observed elevated levels of 53BP1 accumulation at sites of DNA damage (Supplementary Fig. 1e). Moreover, BRCA1, whose recruitment depends on RNF8/RNF168^4^, also accumulated to slightly higher levels in CHD7-depleted cells (Supplementary Fig. 1f), while γH2AX formation was largely unaffected (Supplementary Fig. 1g). Consistent with the dose-dependent nature of RNF8/RNF168-dependent chromatin ubiquitylation for recruitment of downstream factors^17^, 53BP1 accumulation to DSB sites was observed at higher IR doses in CHD7-depleted cells compared to control cells (Supplementary Fig. 1h). Neither the DNA replication-guided recruitment pattern of 53BP1^14^, nor γH2AX foci formation were significantly altered upon CHD7 knockdown (Supplementary Fig. 1h). Collectively, these data suggest that CHD7-dependent chromatin remodeling may impact the DSB response by acting upstream of 53BP1 and curtailing its accumulation at sites of DNA damage.

### CHD7 localizes at DSBs occupied by LIG4 and devoid of 53BP1

**Fig. 2:**
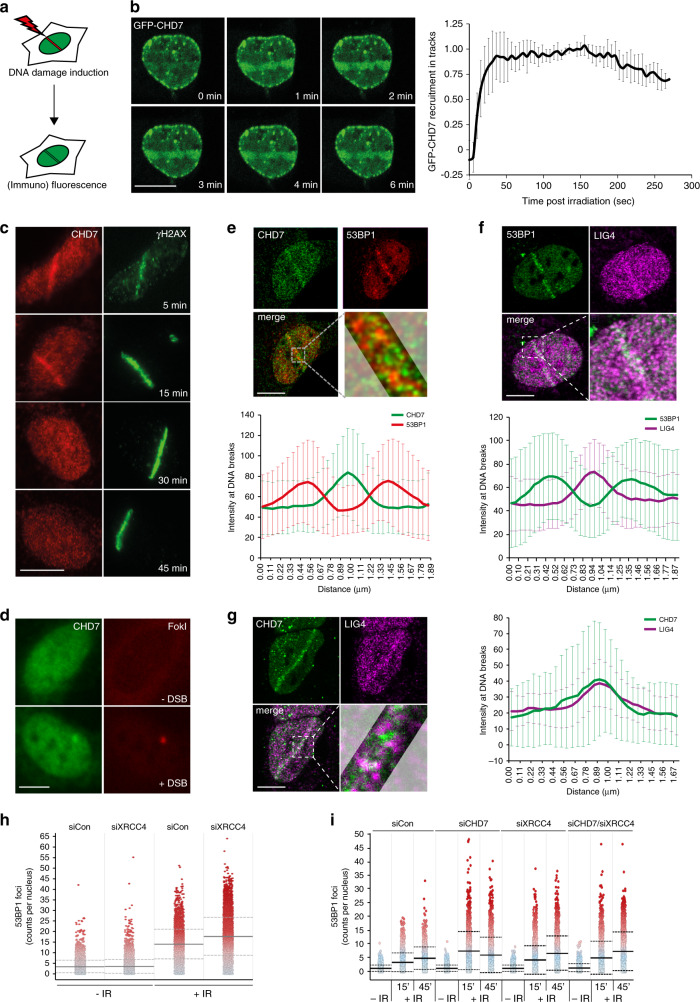
CHD7 localizes at DNA breaks occupied by LIG4 and devoid of 53BP1. **a** Schematic representation of the laser micro-irradiation approach to study protein accumulation at sites of DNA damage. A 365 nm UV-A or 405 nm laser was used on BrdU- or Hoechst-sensitized cells, respectively, whereas an 800 nm multiphoton laser was used on untreated cells. **b** Recruitment of GFP–CHD7 to 800 nm multiphoton tracks in U2OS cells (left panel). Quantification of the data is shown as mean ± SD from seven cells (right panel). **c** Recruitment of endogenous CHD7 to 365 nm UV-A tracks in U2OS cells fixed and stained at different time-points after DNA damage. γH2AX is a DNA damage marker. Representative images from 2 to 5 independent experiments are shown. **d** Endogenous CHD7 recruitment at a stably integrated Lactose operator (LacO) array upon tethering of a Lactose repressor (LacR)-tagged FokI nuclease in U2OS cells treated for 4 h with 1 µM 4-OHT and 0.5 µM shield-1. Representative images from >100 cells of a representative experiment from 3 independent replicates are shown. **e** Colocalization by confocal microscopy of CHD7 and 53BP1 at 365 nm UV-A tracks 15 min after DNA damage induction in U2OS cells (upper panel). Quantification of co-localized foci is shown as mean ± SD from 21 cells acquired in 3 independent experiments (lower panel). **f** As in **e**, except for 53BP1 and LIG4 (upper panel). Quantification of co-localized foci is shown as mean ± SD from 32 cells acquired in at least 3 independent experiments (lower panel). **g** As in **e**, expect for CHD7 and LIG4 (left panel). Quantification of co-localized foci is shown as mean ± SD from 21 cells acquired in at least 3 independent experiments (right panel). **h** Quantification from single-cell QIBC analysis of >1000 cells per condition of 53BP1 accumulation at DSB sites in U2OS cells transfected with the indicated siRNAs. Cells were exposed to 0.5 Gy of IR and foci were quantified after 15 min. Mean (solid line) and standard deviation from the mean (dashed lines) is indicated. **i** As in **h**, but for the indicated single or combined siRNA treatments and with IR-induced 53BP1 foci being quantified after 15 and 45 min. Scale bar 10 µm. Source data are provided as a Source Data file.

To assess whether CHD7 directly impacts the DSB response, we locally inflicted DNA damage using either a multiphoton or UV-A laser (Fig. 2a). GFP–CHD7 was rapidly (within 30 s) recruited to DNA damage tracks induced by multiphoton laser irradiation in U2OS cells (Fig. 2b). Endogenous CHD7 remained associated with these lesions for 15 min, yet was released afterwards and became undetectable at break sites 30–45 min post irradiation (Fig. 2c and Supplementary 2a). We observed accumulation of endogenous CHD7 at UV-A laser-induced DNA damage tracks in G1 and S/G2 phase in U2OS cells (Supplementary Fig. 2b). CHD7 recruitment was not specific to U2OS, as it was also observed in HeLa cells (Supplementary Fig. 2c). Moreover, WT and ATPase-dead (K998R) GFP–CHD7 showed comparable recruitment kinetics, indicating that the chromatin remodeling activity is dispensable for recruitment (Supplementary Fig. 2d). Finally, to examine whether CHD7 is recruited to bona fide DSBs, we monitored its endogenous accumulation at a stably integrated Lactose operator (LacO) array upon tethering of a Lactose repressor (LacR)-tagged FokI nuclease in U2OS cells^21^. CHD7 accumulated at FokI nuclease-induced DNA damage sites (Fig. 2d), corroborating our laser micro-irradiation results.

Although CHD7 and 53BP1 are both present around DNA break sites (Figs. 1b and 2b–d), CHD7 curtails 53BP1 recruitment (Fig. 1 and Supplementary Fig. 1). We therefore tested whether CHD7 and 53BP1 occupy the same chromatin compartment near DSBs. UV-A laser irradiation followed by immunofluorescent co-staining of CHD7 and 53BP1 and analysis of protein intensities along the laser tracks showed that CHD7 and 53BP1 display mutually exclusive localization patterns within the irradiated area (Fig. 2e and Supplementary Fig. 2e). Given that 53BP1 restrains DNA-end resection and is therefore believed to support NHEJ-dependent DSB repair, we compared its localization at DNA damage sites to that of the core NHEJ complex LIG4/XRCC4. Surprisingly, however, 53BP1 and LIG4 did not colocalize in these experiments (Fig. 2f), suggesting that at DSBs 53BP1 is not only spatially distinct from CHD7 (Fig. 2e), but also from core NHEJ factors. This prompted us to also compare the localization of LIG4 and CHD7. Remarkably, we found that CHD7 and LIG4 co-localized near DSBs (Fig. 2g). Given this observation, we assessed whether LIG4/XRCC4, similar to CHD7, curtails 53BP1 accumulation and vice versa. Indeed, 53BP1 foci formation was enhanced in XRCC4-depleted cells (Fig. 2h). Double depletion of CHD7 and XRCC4 did not further increase the number of 53BP1 foci, suggesting that these proteins act epistatically to control 53BP1 accumulation (Fig. 2i). Thus, CHD7 and the core NHEJ factors LIG4/XRCC4 colocalize at DSBs and functionally curtail 53BP1 accumulation (and vice versa), spatially separating their accrual from the chromatin-based assembly of 53BP1.

### PARP1-dependent chromatin relaxation promotes CHD7 recruitment

**Fig. 3:**
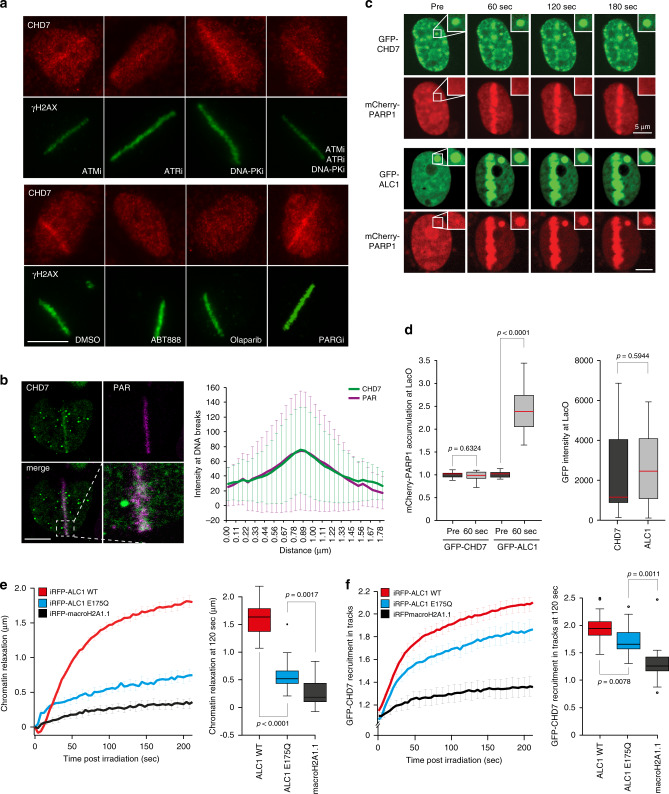
PARP1-depedent chromatin relaxation promotes CHD7 recruitment. **a** CHD7 localization at 365 nm UV-A tracks 15 min after DNA damage induction in U2OS cells treated for 1 h before micro-irradiation with ATM, ATR, DNA-PK inhibitors, PARP inhibitors, and PARG inhibitor. γH2AX is a DNA damage marker. Representative images from 2 to 7 independent experiments are shown. **b** Colocalization by confocal microscopy of CHD7 and PAR chains at 365 nm UV-A tracks 15 min after DNA damage induction in U2OS cells (left panel). Quantification of co-localized foci is shown as mean ± SD from 32 cells acquired in at least 3 independent experiments (right panel). **c** Fluorescence images of in situ PAR-binding three-hybrid assays. GFP–CHD7 or GFP–ACL1 was tethered to a LacO array following co-expression with mCherry-PARP1. DNA damage induction with 405 nm laser micro-irradiation recruited CHD7 and PARP1 to laser tracks. PARP1 co-localized with tethered GFP–ALC1 (positive control), but not GFP–CHD7. Inset shows the magnified LacO array. **d** Quantification of cells from (**c**). The intensity of mCherry signal at the LacO array was quantified pre damage and 60 s post damage (left panel). The intensity of GFP–CHD7 and GFP–ALC1 signals at the LacO array were quantified (right panel). The graphs show the first, median and third quartiles from 14 cells of a representative experiment from among 3 independent replicates. Statistical significance was calculated using the two-tailed Student’s *t* test. **e** Chromatin relaxation was measured in U2OS cells overexpressing iRFP-ALC1 wildtype (WT), iRFP-ALC1 ATPase-dead (E175Q), and iRFP-macroH2A1.1 (left panel). Boxplots show the first, median and third quartiles from 17 to 21 cells of the above data quantified at 120 s post irradiation of a representative experiment from 3 independent replicates (right panel). **f** GFP–CHD7 recruitment to 405 nm laser tracks in U2OS cells overexpressing iRFP-ALC1 wildtype (WT), iRFP-ALC1 ATPase-dead (E175Q), and iRFP-macroH2A1.1 (left panel). Boxplots show the first, median and third quartiles from 17 to 21 cells of the above data quantified at 120 s post irradiation of a representative experiment from 3 independent replicates (right panel). Statistical significance was calculated using the two-tailed Student’s *t* test. Scale bar 10 µm (**a**, **b**), 5 µm (**c**). Source data are provided as a Source Data file.

Next, we sought to address how CHD7 is recruited to DNA breaks. Inhibition of either of the ATM, ATR, or DNA-PK kinases did not have a significant impact on CHD7 recruitment (Fig. 3a and Supplementary Fig. 3a, b). However, treating cells with inhibitors of poly(ADP-ribose) polymerase (PARPi) almost completely abrogated the recruitment of GFP–CHD7 (Supplementary Fig. 3c), as well as endogenous CHD7 (Fig. 3a and Supplementary Fig. 3b, d). Similarly, depletion of PARP1 completely impaired CHD7 recruitment, whereas depletion of PARP2 and PARP3 had no major effect (Supplementary Fig. 3e, f). Conversely, treating cells with poly(ADP-ribose) glycohydrolase inhibitor (PARGi) prolonged CHD7 retention at DNA breaks (Supplementary Fig. 4a, b). Thus, CHD7 is rapidly, but transiently recruited to DSB-containing tracks in a manner dependent on the activity of PARP1.

To further unravel how PARP1 promotes CHD7 accumulation at DNA breaks, we first assessed whether CHD7 and poly(ADP-ribose) (PAR) colocalize at DNA damage sites. We indeed observed colocalization of CHD7 and PAR chains upon laser irradiation (Fig. 3b). To examine whether this involves CHD7’s ability to bind PAR or PARP1, we used a previously established fluorescence three-hybrid assay (Supplementary Fig. 4c)^22^. In contrast to LacO-anchored ALC1, a well-characterized PAR-binding protein that readily recruited PARylated PARP1 that was naturally released from laser-induced DNA damage sites^22,23^, we did not observe an interaction between PARylated PARP1 and LacO-anchored CHD7 (Fig. 3c, d). This suggests that although PARylation is required for CHD7 recruitment, this mode of recruitment may not involve direct PAR binding. Instead, we hypothesized that CHD7 recruitment may depend on PAR-induced chromatin remodeling, as PARP1-dependent PARylation stimulates rapid chromatin relaxation in the vicinity of DNA breaks in a manner dependent on the activity of the CHD2 and ALC1 chromatin remodelers^23,24^. We therefore overexpressed ALC1, which enhances chromatin relaxation after DNA damage, as measured by the change in the width of a photoactivated line corresponding to the damaged area, without affecting PAR signaling (Fig. 3e)^22^. In cells displaying similar level of expression of GFP–CHD7 (Supplementary Fig. 4d), we found that overexpression of WT ALC1 increased CHD7 accumulation at DNA break sites compared to overexpression of ATPase-dead (E175Q) ALC1 (Fig. 3f). Consistently, overexpression of macroH2A1.1, which reduces chromatin relaxation^25^ with a slight, yet statistically not significant impact on PAR chain formation (Supplementary Fig. 4e), reduced CHD7 accrual (Fig. 3e, f). These results suggest that CHD7 recruitment to DNA damage sites requires PARP1-induced chromatin relaxation.

### CHD7 promotes NHEJ repair of DSBs

**Fig. 4:**
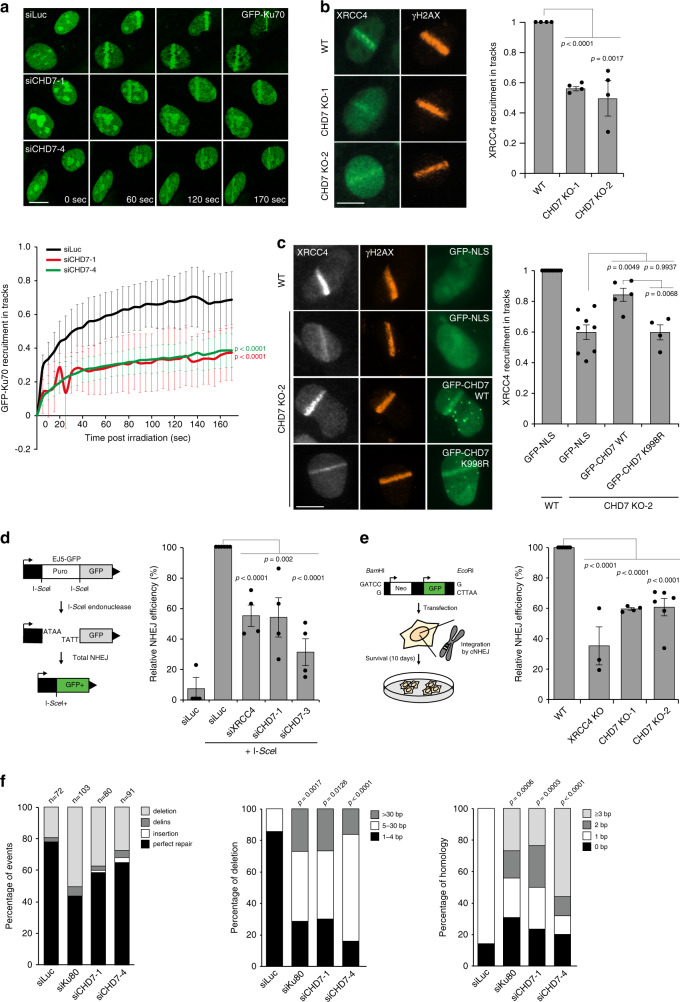
CHD7 promotes NHEJ repair of DSBs. **a** GFP-Ku70 recruitment to 800 nm multiphoton tracks in RPE1-hTERT cells transfected with the indicated siRNAs (upper panel). The mean ± SEM from >100 cells from 4 independent experiments is shown (lower panel). **b** XRCC4 recruitment to 365 nm UV-A tracks 10 min after DNA damage induction in wildtype (WT) and CHD7 knockout (KO) U2OS cells. γH2AX is a DNA damage marker (left panel). The mean ± SEM from >150 cells from 4 independent experiments is shown (right panel). Data were normalized to the WT, which was set to 1. **c** XRCC4 recruitment to 365 nm UV-A tracks 10 min after DNA damage induction in WT and CHD7 KO-2 U2OS cells expressing the indicated GFP fusions. γH2AX is a DNA damage marker (left panel). The mean ± SEM from >80 cells from 4 to 5 independent experiments is shown (right panel). Data were normalized to WT, which was set to 1. **d** Schematic of the EJ5-GFP reporter for NHEJ (left panel). Quantification of EJ5-GFP-positive U2OS cells transfected with the indicated siRNA and I-*Sce*I expression vector. I-*Sce*I transfection was corrected by co-transfection with mCherry expression vector. The mean ± SEM of 4 independent experiments is shown (right panel). Data were normalized to siLuc, which was set to 100%. **e** Schematic of the plasmid integration assay (left panel). Quantification of plasmid integration efficiencies in WT and the indicated U2OS KO cells (right panel). The mean ± SEM from 3 to 6 independent experiments is shown. Data were normalized to WT, which was set to 100%. (**f**) Mutational signatures (left panel), deletion sizes (middle panel) and microhomology usage (in case of deletion formation) (right panel) at repair junctions in cells containing the GC92 reporter for NHEJ transfected with the indicated siRNAs and I-*Sce*I expression vector. The bars represent data obtained from 3 independent experiments. Statistical significance for microhomology usage was calculated for sequences showing at least 1 bp of microhomology. Statistical significance was calculated using the two-tailed Student’s *t* test (all panels except of (**f**)) or the two-tailed Mann–Whitney *U* test (panel **f**). Scale bar 10 µm. Source data are provided as a Source Data file.

The PARP1-dependent recruitment of CHD7 (Fig. 3a), its colocalization with LIG4 (Fig. 2g) and PAR chains (Fig. 3b), as well as the fact that PARP1 promotes XRCC4 recruitment and NHEJ at DNA breaks^24^ encouraged us to investigate whether CHD7 supports NHEJ. We first assessed whether CHD7 affects recruitment of the NHEJ factor Ku70. To this end, cells expressing endogenously GFP-tagged Ku70^26^ were depleted of CHD7 and subjected to multiphoton laser micro-irradiation. Strikingly, CHD7 depletion reduced GFP-Ku70 accumulation when compared to that in control cells (Fig. 4a and Supplementary Fig. 5a). In agreement with this observation, we also found that the accumulation of endogenous XRCC4, which operates downstream of Ku70/80^3^, was reduced at UV-A laser-induced DNA damage sites in two independent CHD7 knockout U2OS clones (Fig. 4b and Supplementary Fig. 5b). Importantly, we were able to partially complement the reduced XRCC4 recruitment by re-expression of GFP-tagged WT, but not ATPase-dead (K998R) CHD7 (Fig. 4c and Supplementary Fig. 5c). Taken together, these results indicate that CHD7 chromatin remodeling supports the recruitment of NHEJ factors to DSBs.

To test whether the reduced loading of cNHEJ factors in CHD7-deficient cells impairs NHEJ-mediated DSB repair, we used the well-established EJ5-GFP reporter assay (Fig. 4d). Flow cytometric analysis of GFP fluorescence revealed that NHEJ was reduced following CHD7 depletion similar to that after XRCC4 depletion (Fig. 4d). The EJ5-GFP reporter provides a readout for total NHEJ activity, including both cNHEJ and altNHEJ^27^. To address if CHD7 specifically affects Ku70/80- and XRCC4-dependent cNHEJ, we used random plasmid integration into genomic DNA as a measure (Fig. 4e)^24^. Indeed, depletion or knockout of CHD7 impaired cNHEJ, as did knockout of XRCC4 or depletion of Ku80 and DNA-PK. As expected, BRCA2 depletion had no effect (Fig. 4e and Supplementary Fig. 5d, f).

Impaired cNHEJ shifts DSB repair to altNHEJ as evidence by a reduced accurate end-joining concomitantly with a switch to error-prone repair due to microhomology use^3^. To test whether impaired cNHEJ in CHD7-depleted cells impacts the mutational signature at repair junctions, we used a previously published NHEJ reporter in GC92 cells from which repair junctions can be amplified and Sanger-sequenced (Supplementary Fig. 5g)^28^. Ku80 depletion led to an increase in the formation of larger deletions and to the usage of larger stretches of microhomology (Fig. 4f and Supplementary Fig. 5h)^3^. Interestingly, CHD7 depletion also increased the proportion of larger deletions and use of microhomology during repair (Fig. 4f and Supplementary Fig. 5h), further supporting the involvement of CHD7 in cNHEJ.

To study the mutagenic nature of DSB repair in the absence of CHD7 in more detail, we employed the well-established EJ2-GFP reporter for altNHEJ (Supplementary Fig. 5i)^27^. LIG3 depletion impaired altNHEJ, as expected given its known role in this repair process^29^. In contrast, CHD7 depletion did not impair altNHEJ, but instead enhanced the efficiency of this repair process. Finally, co-depletion of LIG3 and CHD7 impaired altNHEJ to similar levels as observed after LIG3 depletion alone (Supplementary Fig. 5j, k). Together, the results obtained with these different assays suggest that CHD7 is not required for altNHEJ, but rather promotes cNHEJ.

Since the loss of microhomology-mediated end joining by PolQ was reported to be synthetic lethal with BRCA1/2 deficiency^30,31^, we aimed at further substantiating our findings by unraveling how CHD7 genetically interacts with PolQ and BRCA2. Loss of either CHD7, PolQ, or BRCA2 alone moderately impacted cell survival. However, loss of both PolQ and BRCA2 further impaired cell survival as previously published^30,31^. Strikingly, while loss of both CHD7 and PolQ led to a statistically insignificant decrease in clonogenic survival, the combined loss of CHD7 and BRCA2 significantly decreased the cloning efficiencies (Supplementary Fig. 5l). Further corroborating these findings, we found that CHD7 loss, similar to XRCC4 depletion, impairs clonogenic survival of VH10–SV40-immortalized fibroblasts following induction of IR-induced DSBs (Supplementary Fig. 5m, n). The increased IR sensitivity likely resulted from an accumulation of unresolved DSBs, as suggested by slightly increased γH2AX foci numbers following IR-exposure of CHD7-depleted G1 cells (Supplementary Fig. 6a, b). Furthermore, the shift from cNHEJ towards error-prone altNHEJ after CHD7 depletion can increase mutagenic DSB repair, which may impact cell survival as well (Fig. 4f, and Supplementary Fig. 5h, j). Taken together, these results demonstrate that CHD7 contributes to the accumulation of cNHEJ factors and promotes end-joining-dependent repair of DSBs.

The loss of CHD7 did not affect the expression of NHEJ proteins as revealed by RNA-sequencing (Supplementary Fig. 6c and Supplementary Data 2) and western blot analysis (Supplementary Fig. 6d). Furthermore, CHD7 loss has been shown to upregulate the expression of p53 (Supplementary Fig. 6e)^32^, which has been implicated in cell cycle control and DSB repair^33^. However, increased levels of p53 in CHD7-deficient cells neither impacted cell cycle progression (Supplementary Fig. 6f), nor the accumulation of XRCC4 (Supplementary Fig. 6g). These results suggest a direct, rather than an indirect role for CHD7 in DSB repair via NHEJ.

Finally, we asked if CHD7 plays a unique role in NHEJ, or also affects HR. We first compared its localization at DNA break sites with the core HR factor BRCA1, but did not observe colocalization for these proteins (Supplementary Fig. 7a). Next, we used the well-established DR-GFP reporter assay (Supplementary Fig. 7b), which revealed that knockout of CHD7, in contrast to BRCA1 depletion, did not impair HR (Supplementary Fig. 7c, d). We also assessed whether CHD7 loss would render cells sensitive to PARP inhibition, which is a feature of HR-deficient cells^2^. However, we did not observe increased sensitivity to PARPi treatment of CHD7 knockout cells, as opposed to that of BRCA2-depleted cells (Supplementary Fig. 7e). Finally, we found that CHD7 did not affect foci formation of the core HR factor RAD51 in S-phase cells (Supplementary Fig. 7f). Taken together, our results suggest that CHD7 promotes DSB repair by NHEJ, but not by HR.

### CHD7 associates with HDAC1 and recruits it to DNA breaks

**Fig. 5:**
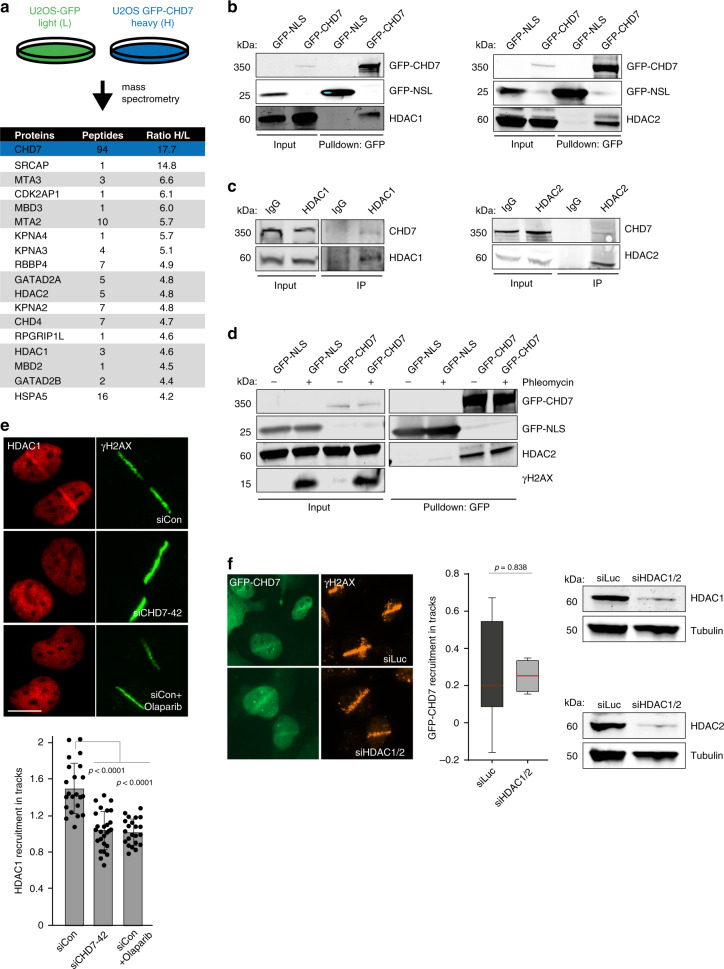
CHD7 recruits HDAC1 to DNA breaks. **a** SILAC-based mass spectrometry analysis of U2OS cells transiently expressing GFP (L) or GFP–CHD7 (H). NuRD complex members are marked in dark gray. **b** Pulldowns of the indicated GFP fusion proteins in U2OS cells. Blots were probed for GFP, HDAC1, and HDAC2. Representative blots from 3 independent experiments are shown. **c** Immunoprecipitation (IP) of endogenous HDAC1 and HDAC2 in U2OS cells. IgG is a control IP. Blots were probed for CHD7, HDAC1, and HDAC2. Representative blots from 2 independent experiments are shown. **d** Pulldowns of the indicated GFP fusion proteins in U2OS cells treated for 1 h with 500 µM phleomycin. Blots were probed for GFP, HDAC2, and γH2AX. Representative blots from 2 independent experiments are shown. **e** HDAC1 recruitment to 365 nm UV-A tracks 15 min after DNA damage induction in U2OS cells transfected with the indicated siRNAs and treated for 1 h with the PARP inhibitor olaparib before micro-irradiation. γH2AX is a DNA damage marker (upper panel). The mean ± SD from 22 to 26 cells from at least 3 independent experiments is shown (lower panel). Statistical significance was calculated using the two-tailed Student’s *t* test. **f** GFP–CHD7 recruitment to 365 nm UV-A tracks 5 min after damage induction in U2OS cells transfected with indicated siRNAs (left panel). Boxplots show the first, median and third quartiles from at least 3 independent experiments (middle panel). Statistical significance was calculated using the two-tailed Student’s *t* test. Western blot analysis of HDAC1 and HDAC2 expression. Tubulin is a loading control (right panel). Scale bar 10 µm. Source data are provided as a Source Data file.

To further elucidate the role of CHD7 in NHEJ, we aimed to identify proteins that interact with CHD7. We transiently expressed GFP-tagged CHD7 in U2OS cells and performed GFP–CHD7 pulldowns followed by mass spectrometry (MS) after stable isotope labeling by amino acids in culture (SILAC) (Supplementary Data 3). The top interactors were components of the NuRD chromatin remodeling complex (Fig. 5a), which was previously implicated in DSB repair^34–37^. Interestingly, among these components were the histone de-acetylases HDAC1 and HDAC2. GFP pulldowns coupled to western blot analysis confirmed that GFP-tagged CHD7 interacted with endogenous HDAC1 and HDAC2 (Fig. 5b), while reciprocal pulldowns of endogenous HDAC1 and HDAC2 revealed interactions with endogenous CHD7 (Fig. 5c). These interactions were DNA damage-independent (Fig. 5d), suggesting that CHD7 and HDAC1/2 form a protein complex prior to DSB induction.

HDAC1/2 were previously shown to promote NHEJ^35,38^. To investigate a potential interplay between CHD7 and HDAC1 during NHEJ, we analyzed whether CHD7 recruits HDAC1 to sites of DNA damage. HDAC1 recruitment to UV-A laser-induced DNA damage was completely abolished in cells depleted for CHD7, as well as in cells treated with PARP inhibitor (Fig. 5e and Supplementary Figs. 6d and 8a). On the contrary, recruitment of GFP-tagged CHD7 to UV-A laser-induced DNA damage was independent of HDAC1/2 (Fig. 5f and Supplementary Fig. 6d), suggesting that HDAC1 recruitment depends on CHD7, but not vice versa.

### CHD7 and HDAC1 regulate DNA damage-induced chromatin dynamics

**Fig. 6:**
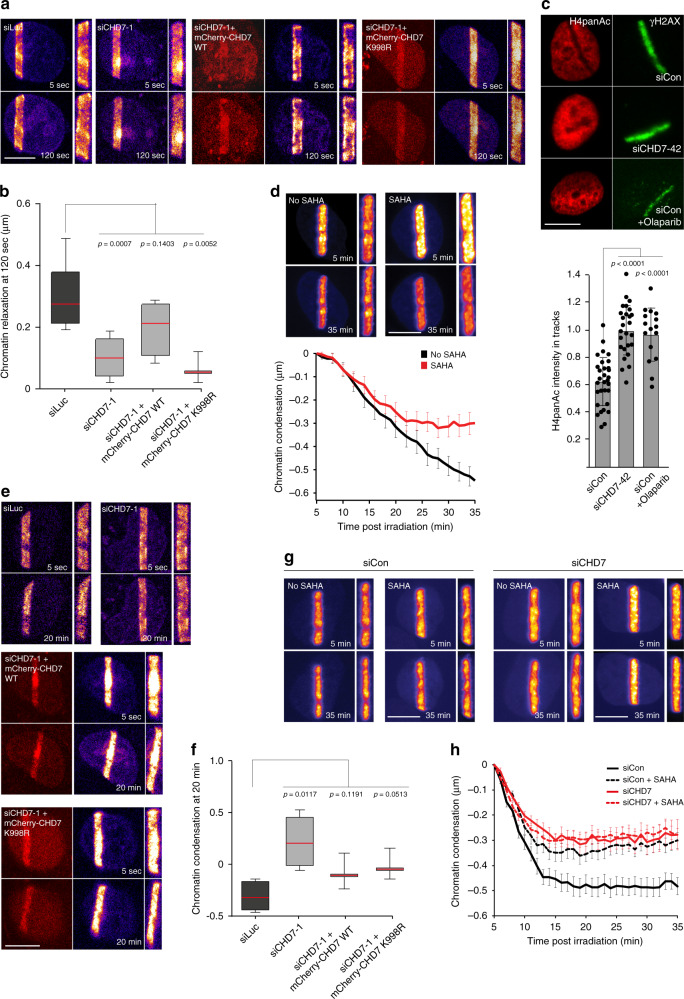
CHD7 and HDAC1 regulate DNA damage-induced chromatin dynamics. **a** Chromatin relaxation in U2OS cells as measured by the thickness of the photoactivated PAGFP-H2A area in cells transfected with the indicated siRNAs and expressing the indicated mCherry fusions prior to 800 nm multiphoton laser micro-irradiation. **b** Quantification of chromatin relaxation at 120-s post irradiation from (**a**). Boxplot shows the first, median and third quartiles from >40 cells from 3 to 7 independent experiments. **c** H4 de-a**c**etylation at 365 nm UV-A tracks 15 min after DNA damage induction in U2OS cells transfected with indicated siRNAs and treated for 1 h with the PARP inhibitor olaparib before micro-irradiation. γH2AX is a DNA damage marker (upper panel). The mean ± SD from 16 to 31 cells per condition is shown (lower panel). **d** Chromatin condensation in U2OS cells as measured up to 35 min post irradiation by the thickness of the photoactivated PAGFP-H2B area in cells treated for 5 min with the HDAC inhibitor SAHA prior to 405 nm laser micro-irradiation (upper panel). Quantification of chromatin condensation is presented as the mean ± SEM of 28 cells per condition from a representative of 3 independent experiments (lower panel). **e** Chromatin condensation in U2OS cells transfected with the indicated siRNAs and expressing the indicated siRNA-resistant mCherry fusions as measured up to 20 min post irradiation by the thickness of the photoactivated PAGFP-H2A area prior to 800 nm multiphoton laser micro-irradiation. **f** Quantification of chromatin condensation at 20 min post irradiation from e. Boxplot shows the first, median and third quartiles from >40 cells from 3 to 4 independent experiments. **g** Chromatin condensation in U2OS cells as measured up to 35 min post irradiation by the thickness of the photoactivated PAGFP-H2B area in cells treated with the indicated siRNAs, and for 5 min with the HDAC inhibitor SAHA prior to 405 nm laser micro-irradiation. **h** Quantification of chromatin condensation from **g** is presented as the mean ± SEM of 20–25 cells per condition from a representative of 3 independent experiments. Statistical significance was calculated using the two-tailed Student’s *t* test (all panels). Scale bar 10 µm. Source data are provided as a Source Data file.

Previous studies^23,24,39–41^ reported a rapid PARP1-dependent expansion of chromatin at sites of DNA damage. Given that CHD7 and HDAC1 are recruited to DNA breaks in a manner dependent on PARP1 activity and that they modify chromatin structure, we asked whether their loss would affect chromatin expansion. To examine this possibility, we expressed histone H2A or H2B fused to photoactivable GFP and activated PAGFP following laser micro-irradiation to monitor chromatin changes in DNA damage-containing tracks by determining their absolute width (Supplementary Fig. 8b). Acute HDAC inhibition with trichostatin A (TSA) and suberoylanilide hydroxamic acid (SAHA) did not affect chromatin expansion (Supplementary Fig. 8c). However, depletion of CHD7 substantially reduced expansion, and this effect was rescued by re-expression of mCherry-tagged WT, but not ATPase-dead (K998R) CHD7 (Fig. 6a, b and Supplementary Fig. 8d).

To study the relevance of the CHD7–HDAC1 interaction and colocalization at DNA breaks, we analyzed the histone H4 acetylation status following UV-A laser micro-irradiation. We found that DNA damage induction leads to local histone H4 de-acetylation as revealed by the formation of an anti-stripe (Fig. 6c and Supplementary Fig. 8e), in agreement with previous reports^35^. Acute HDAC inhibition prior to DNA damage induction using three different HDAC inhibitors, TSA, SAHA or Romidepsin, completely abrogated histone H4 de-acetylation (Supplementary Fig. 8e). Interestingly, histone H4 de-acetylation at DNA breaks was also impaired in CHD7-depleted, as well as in PARPi-treated cells (Fig. 6c). Together our results suggest that CHD7 is an integral part of a PARP1-initiated and HDAC1/2-executed signaling axis that drives histone H4 de-acetylation at DNA breaks.

Following an initial rapid expansion of chromatin at sites of DNA damage, recondensation of the damaged chromatin is observed within 15–30 min after DNA damage induction^23,42^. We hypothesized that the local de-acetylation of damaged chromatin via HDAC1/2 downstream of CHD7 recruitment may participate in this chromatin recondensation process. Indeed, we found that although HDAC inhibition did not affect the rapid expansion of damaged chromatin (Supplementary Fig. 8c), it partially inhibited chromatin recondensation (Fig. 6d and Supplementary Fig. 8f, g). Similarly, we observed that CHD7 depletion not only inhibited early chromatin relaxation (Fig. 6a, b and Supplementary Fig. 8d), but also impaired the recondensation process as revealed by more persistent chromatin expansion in CHD7-depleted cells 20 min after irradiation (Fig. 6e, f and Supplementary Fig. 8h, i). Of note, while the involvement of CHD7 in chromatin relaxation was dependent on its remodeling activity (Fig. 6a, b and Supplementary Fig. 8d), we found that the impairment in chromatin recondensation in CHD7-depleted cells could be reversed by re-expression of mCherry-tagged CHD7 WT and ATPase-dead (K998R) (Fig. 6e, f and Supplementary Fig. 8h, i). This suggests that the re-compaction of damaged chromatin is not dependent on CHD7’s chromatin remodeling activity, but rather depends on its role in recruiting HDAC1/2 to DNA breaks via a physical interaction. Consistently, pulldown experiments revealed that both WT and ATPase-dead (K998R) GFP-tagged CHD7 interact with HDAC1 to comparable levels (Supplementary Fig. 9a). Moreover, simultaneous CHD7 depletion and HDAC inhibition via SAHA had an epistatic effect on the recondensation process (Fig. 6g, h). Taken together, these results suggest a dual role for CHD7 in that it promotes rapid expansion of damaged chromatin in a manner dependent on its ATPase activity and subsequently triggers chromatin re-compaction by recruiting HDAC1/2 de-acetylation activities. In line with this, we observed that HDAC1, similar to CHD7, recruits to laser-induced DNA damage sites already within 5 min. However, contrary to CHD7, but in agreement with a role in the time-delayed chromatin compaction process, HDAC1 remained associated with DNA damage up to 30 min post irradiation (Supplementary Fig. 9b).

HDAC1/2-dependent chromatin de-acetylation is known to act as a suppressive modification that affects transcription^43^. Interestingly, transcription repression has been shown to occur at DNA breaks, where it facilitates NHEJ by impacting the binding of NHEJ factors^44,45^. We therefore investigated whether CHD7 and HDAC1/2 affect NHEJ by promoting transcription silencing at DNA breaks using two established approaches. First, we examined the levels of nascent transcripts using 5-ethynyl uridine (EU) incorporation at sites of DNA damage inflicted by UV-A laser micro-irradiation (Supplementary Fig. 9c)^46^. Second, we employed a well-established DSB reporter system to study transcription activity around DSBs (Supplementary Fig. 9d)^21^. However, neither CHD7 loss nor HDAC1/2 depletion significantly affected transcriptional silencing at DNA breaks in these assays (Supplementary Fig. 9e–h). Thus, it seems unlikely that CHD7 and HDAC1/2 impact NHEJ indirectly by regulating transcription repression at DSBs.

**Fig. 7:**
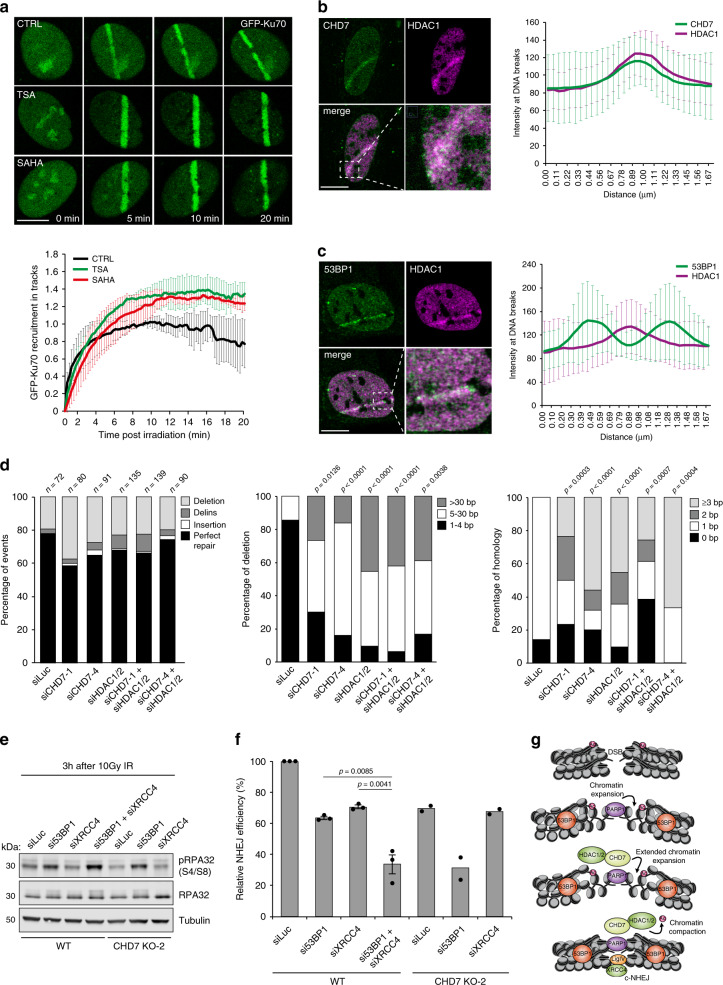
CHD7-HDAC1/2 and 53BP1 synergistically affect NHEJ. **a** GFP-Ku70 recruitment to tracks in RPE1-hTERT cells treated for 5 min with the HDAC inhibitors TSA and SAHA before 800 nm multiphoton micro-irradiation (upper panel). The mean ± SEM from >60 cells from 2 independent experiments is shown (lower panel). **b** Colocalization by confocal microscopy of CHD7 and HDAC1 at 365 nm UV-A tracks 15 min after damage induction in U2OS cells (left panel). Quantification of co-localized foci is shown as mean ± SD from 31 cells acquired in at least 3 independent experiments (right panel). **c** As in **b**, except for 53BP1 and HDAC1 (left panel). Quantification of co-localized foci is shown as mean ± SD from 17 cells acquired in at least 3 independent experiments (right panel). **d** Mutational signatures (left panel), deletion sizes (middle panel) and microhomology usage (in case of deletion formation) (right panel) at repair junctions in the GC92 reporter for NHEJ. GC92 cells were transfected with the indicated siRNAs and I-*Sce*I expression vector. Repair junctions were amplified by PCR and Sanger-sequenced. The bars represent data obtained from 3 independent experiments. Statistical significance was calculated using the two-tailed Mann–Whitney *U* test. Statistical significance for microhomology usage was calculated for sequences showing at least 1 bp of microhomology. The junctions of the siLuc, siCHD7-1, and siCHD7-4 samples are the same as those presented in Fig. 4f. **e** Western blot analysis of phosphorylated RPA32 (S4/S8) and RPA32 expression in WT and CHD7 KO-2 U2OS cells transfected with the indicated siRNAs. Cells were examined 3 h after 10 Gy of ionizing radiation. Tubulin is a loading control. Representative blots from 2 independent experiments are shown. **f** Quantification of plasmid integration efficiencies in WT and CHD7 KO-2 U2OS cells transfected with the indicated siRNAs. The mean ± SEM from 2 to 3 independent experiments is shown. Data were normalized to WT, which was set to 100%. Statistical significance was calculated using the two-tailed Student’s *t* test. **g** Model for how the CHD7-HDAC1/2-dependent chromatin remodeling promotes DSB repair by error-free cNHEJ. Scale bar 10 µm. Source data are provided as a Source Data file.

Strikingly, when we monitored the NHEJ protein Ku70 at DNA breaks following HDAC1/2 inhibition, we observed an increase in GFP-Ku70 recruitment in TSA- and SAHA-treated cells (Fig. 7a), consistent with previous work^35^. Moreover, reduced GFP-Ku70 recruitment to DNA damage sites observed in CHD7-depleted cells (Fig. 4a) was partially rescued by HDAC1/2 inhibition via SAHA (Supplementary Fig. 10a). To test whether the increase in Ku70 levels upon HDAC1/2 inhibition resulted from impaired chromatin recondensation, we monitored GFP-Ku70 loading at DNA breaks in cells bathed with hypotonic medium. Such medium induces global chromatin decondensation^47^ and impairs chromatin recondensation consecutive to damage induction (Supplementary Fig. 10b). We found that hypotonic treatment is sufficient to induce over-accumulation of GFP-Ku70, although not to the same level as HDAC1/2 inhibition via TSA (Supplementary Fig. 10c). Nevertheless, combining both treatments had no further impact on GFP-Ku70 recruitment, suggesting that the over-accumulation of GFP-Ku70 is mostly caused by impaired chromatin recondensation (Supplementary Fig. 10c). Together our findings suggest that while CHD7-dependent expansion of damaged chromatin promotes recruitment of NHEJ factors at DNA breaks, the subsequent HDAC1/2-dependent re-compaction of the damaged chromatin restrains the accumulation of these repair factors.

### CHD7–HDAC1/2 and 53BP1 synergistically affect NHEJ

Our results provide evidence that CHD7 and HDAC1/2 jointly support DSB repair via NHEJ. Indeed, we found that CHD7 and HDAC1 colocalize at DNA break sites (Fig. 7b), while HDAC1, similar to CHD7, did not colocalize with 53BP1 (Fig. 7c). Thus, CHD7 and HDAC1 may operate in the same chromatin domains near DSBs in manner distinct from 53BP1. To corroborate this point, we analyzed mutational signatures at repair junctions in the NHEJ reporter of CHD7- and/or HDAC1/2-depleted GC92 cells^28^. CHD7 or HDAC1/2 depletion increased the proportion of deletions at repair junctions and these deletions were generally larger than in control cells (Fig. 7d). Moreover, repair junctions from CHD7 or HDAC1/2-depleted cells showed increased usage of 2 or more nucleotides microhomology (Fig. 7d). Interestingly, triple knockdown of CHD7 and HDAC1/2 did not further increase the proportion of deletions or usage of microhomology (Fig. 7d), indicating that these proteins function epistatically during DSB repair via cNHEJ and support error-free re-ligation of broken DNA ends.

53BP1 constrains the resection of broken ends, thereby stimulating NHEJ^5^. Unclear is whether CHD7–HDAC1/2 act in a similar manner to promote error-free NHEJ. We therefore examined their effect on the processing of IR-induced DSBs using phosphorylated RPA32 at serine 4 and serine 8 (pRPA) as a read-out. We observed increased pRPA levels in 53BP1-depleted cells as was shown previously (Fig. 7e and Supplementary Fig. 10d, e)^6^. In line with the increased 53BP1 accumulation at DSBs in CHD7 and XRCC4 knockdown cells (Figs. 1d, e and 2h, i), we detected slightly reduced pRPA levels in these cells (Fig. 7e and Supplementary Fig. 10d, e). Double depletion of CHD7 and XRCC4 did not further affect pRPA formation. However, the simultaneous loss of 53BP1 and XRCC4, or 53BP1 and CHD7 substantially increased pRPA levels (Fig. 7e and Supplementary Fig. 10d, e). Finally, we assessed the effect of CHD7 or XRCC4 loss alone, or their loss in combination with 53BP1, on cNHEJ in random plasmid integration assays. Consistently with our previous results (Fig. 7e), we found that the cNHEJ defect observed after CHD7 loss was epistatic with that observed after XRCC4 depletion (Fig. 7f). In contrast, cNHEJ was more severely compromised when both CHD7 and 53BP1, or XRCC4 and 53BP1 were lost (Fig. 7f), We therefore propose that 53BP1 is part of a “fail-safe” mechanism that restrains DNA end-resection to promote minimally mutagenic NHEJ when CHD7–HDAC1/2-dependent error-free cNHEJ fails (Fig. 7g and Supplementary Fig. 10f). Consequently, loss of both pathways unleashes extensive end-resection, which at one point becomes incompatible with re-ligation of the broken DNA ends, promoting erroneous repair (e.g., via single-strand annealing) (Fig. 7g and Supplementary Fig. 10f)^48^.

## Discussion

In this study, we identify the chromatin remodeler CHD7 as novel component of the DSB repair machinery and establish that a progressive reorganization of damaged chromatin through the activities of CHD7 and HDAC1/2 supports cNHEJ and error-free re-ligation of broken DNA ends (Fig. 7g and Supplementary Fig. 10f). CHD7 is the protein most frequently mutated in CHARGE syndrome, with the great majority of CHD7 mutations resulting in loss of function^18^. Given that several human developmental disorders are linked to defective genome integrity maintenance^1^, it is possible that erroneous DNA repair plays a thus far unappreciated role in CHARGE etiology. Further studies would be needed to address this point and test for signs of DNA damage in CHARGE patients.

CHD7 recruits to DNA damage sites in a manner dependent on the activity of PARP1. The recruitment of several other chromatin remodelers, including ALC1 and CHD2, has also been shown to be dependent on PARP1 activity. More specifically, these chromatin remodelers appeared to contain PARP1/PAR-binding domains that mediate the interaction with PARP1-induced PAR moieties at sites of DNA damage^24,49^. CHD7, however, does not contain any of the canonical PARP1/PAR-binding domains. Consistently, we did not detect any physical interaction between CHD7 and PARP1/PAR, suggesting that CHD7 recruitment relies on a mechanism that is independent of direct PARP1/PAR-binding. Indeed, we found that CHD7 recruitment occurs in a manner dependent on the PARP1-induced relaxation of damaged chromatin. A similar dependency has been recently observed for the recruitment of the CHD3 and CHD4 chromatin remodelers to damaged chromatin^22^. Thus, PARP1-dependent chromatin relaxation creates a local environment at sites of DNA damage that is amenable to the recruitment of chromatin remodelers and potentially other proteins involved in the DNA damage response.

We demonstrate that a PARP1–CHD7–HDAC1/2 axis drives rapid expansion of damaged chromatin followed by prolonged recondensation to promote NHEJ. Chromatin re-compaction via macroH2A1 and PRDM2 has previously been reported to promote HR^42^. This raises the question as to how condensed chromatin supports both NHEJ and HR. The histone variant macroH2A1 and PRDM2 generate the repressive H3K9me2 mark, while HDAC1/2 on the other hand repress chromatin by de-acetylating lysine residues in the H3 and H4 N-terminal tails. These differential chromatin changes likely reflect the binding of HR and NHEJ factors, respectively. Indeed, a previous report showed that the HR factor BRCA1 associates with condensed chromatin through binding to H3K9me2 or unmodified H3K4^42^. In contrast, H3/H4 acetylation promotes Ku recruitment to DSB sites^35,50^. Thus, condensed chromatin on the one hand allows for accrual of HR factors at DSBs, while on the other hand it prevents supraphysiological levels of NHEJ proteins at DSBs. We hypothesize that condensed chromatin may increase the off-rates of cNHEJ factors at sites distal to DSBs, thereby promoting the release of cNHEJ factors and preventing their spreading away from the break site. This may in turn restrict the binding of cNHEJ factors to broken ends that undergo re-ligation, thereby promoting cNHEJ. While cNHEJ dominates DSB repair in G1-phase cells, it competes with HR in G2-phase cells, and if cNHEJ fails, slow repair by HR comes in place^51^. This pathway requires chromatin re-compaction, not only to promote the accumulation of HR factors, but also to prevent the further accumulation of cNHEJ factors. Thus, a time-dependent change in chromatin conformation at DSBs may not only be critical for the actual repair by NHEJ and HR, but may also dictate a proper shift from NHEJ to HR.

Somewhat unexpectedly, we found that NHEJ-associated processes can suppress 53BP1 assembly on chromatin surrounding DSBs. 53BP1 plays an important role in restraining DNA end-resection, thus shifting the balance from HR towards NHEJ^5,6^. How can this be reconciled with excessive 53BP1 accumulation when NHEJ is inefficient? 53BP1 displays rather late recruitment kinetics (10–15 min following DNA damage induction)^52^ as compared to core cNHEJ factors such as Ku or LIG4/XRCC4 (appear at DNA breaks within seconds)^24^. Furthermore, 53BP1 loss in DT40 cells causes milder radiosensitivity when compared to that of Ku70-deficient cells, and 53BP1 is involved only in a subset of V(D)J recombination events^53^. Moreover, the recent discovery of the Shieldin complex as downstream effector of 53BP1 suggests that 53BP1, rather than acting at clean DSBs, may require a ssDNA overhang for its function to limit DNA end-resection^6^. In line with this, our experiments indicate that 53BP1 and cNHEJ factors such as LIG4/XRCC4 do not occupy the same DSB compartment and do not show epistasis in cNHEJ. We also did not observe colocalization or epistasis between 53BP1 and CHD7/HDAC1, although the latter two promote NHEJ. These results suggest that the rapid repair of clean DSBs via Ku-LIG4/XRCC4-dependent error-free cNHEJ may not strictly rely on 53BP1. However, more complex DSBs, which cannot be easily re-ligated, may be repaired with slower kinetics as they undergo limited DNA end processing. These DSBs may be the preferred substrates for 53BP1–Shieldin to act upon. Accordingly, 53BP1–Shieldin would be needed to restrain DNA end-resection of such breaks and still channel the repair towards (now error-prone) NHEJ. Consistently, impaired error-free NHEJ of clean DSBs, e.g., in absence of CHD7 or core NHEJ factors, may induce limited DNA end-resection, which then needs to be controlled by 53BP1-Shieldin. Loss of CHD7, which contains a methylation mark-binding chromodomain, may also alleviate competition for H4K20me2 binding, and thereby additionally foster 53BP1 recruitment. When 53BP1–Shieldin functions are also lost, such breaks undergo further resection and are a substrate for erroneous repair^54^. In line with that, recent work demonstrated that 53BP1, albeit in the context of HR, promotes error-free gene conversion by restraining excessive DNA end-resection, thereby limiting mutagenic DSB repair by single-strand annealing^48^. Thus, 53BP1–Shieldin accumulation, rather than being a core component of one or the other DSB repair pathway, may tune the extent of DNA end-resection in a break- and context-dependent manner. Accordingly, the role of CHD7 within the PARP1–CHD7–HDAC1 axis described in this study likely drives efficient NHEJ repair by promoting quick and error-free re-ligation of broken DNA ends through Ku and LIG4/XRCC4 activities. If this pathway fails, reinforced 53BP1 assembly at the damaged chromatin protects DNA ends from extensive resection and thereby reinforces end-joining repair of these lesions, albeit at the cost of mutagenic loss of sequence information. With CRISPR-mediated genome engineering currently taking center stage, understanding the balance between error-free and error-prone NHEJ repair may be important to guide precision genome editing.

## Methods

### Cell lines

Human HEK293T, VH10-SV40, RPE1-hTERT, HeLa, U2OS, and SV40 T-transformed GM639 human fibroblasts were cultured in 5% CO_2_ at 37 °C in DMEM (Dulbecco’s modified Eagle’s medium) supplemented with 10% fetal calf serum and antibiotics. RPE1-hTERT cells expressing endogenous GFP-Ku70 were a gift from Steve Jackson^26^. U2OS cells with stably integrated EJ5-GFP, EJ2-GFP, or DR-GFP reporters were a gift from Jeremy Stark and Maria Jasin^27,55^. U2OS cells stably expressing cell cycle markers mKO-Cdt1 and mCherry-geminin were previously generated^24^. SV40 T-transformed GM639 human fibroblasts with a stably integrated GC92 reporter were a gift from Bernard Lopez^28^. U2OS-2B2 cells carrying a genomically integrated LacO array for use in the fluorescence three-hybrid assay were generated previously^56^. U2OS cells stably expressing H2B-PAGFP were described previously^22^. U2OS 2-6-3 cells stably expressing ER-mCherry-LacR-FokI-DD were a gift from Roger Greenberg^21^. U2OS cells expressing a doxycycline-inducible shRNA against RNF168 were a kind gift from Jiri Lukas^17^. CHD7 and XRCC4 knockout U2OS cells were generated by co-transfection of pKLV-U6gRNA-EF(BbsI)-PGKpuro2ABFP (Addgene) containing CHD7 gRNA-1 (5′-GTGACTCACTATCTGGTGAA-3′), CHD7 gRNA-2 (5′-GAACACAAAGTGCTGCTGAC-3′), or XRCC4 gRNA (5′-GATGACATGGCAATGGAAA-3′) with pSpCas9(BB)-2A-GFP (PX458) containing Cas9 (Addgene). Forty-eight hour post transfection cells were sorted by flow cytometry for BFP and GFP expression, seeded at low density after which individual clones were isolated. Knockout of CHD7 and XRCC4 was first verified by Sanger sequencing and TIDE analysis. Clones harboring out-of-frame deletions in *CHD7* and *XRCC4* were further verified by western blot analysis.

### Chemicals

The PARP inhibitors ABT888 (Selleck Chemicals) and olaparib (Selleck Chemicals) were used at a final concentration of 1–10 μM. PARG (PDD00017273; Sigma), ATM (KU-55933; Selleck Chemicals), ATR (AZ-20; Tocris), and DNA-PK (NU-7441; Selleck Chemicals) inhibitors were used at a final concentration of 10 μM. The HDAC inhibitors TSA (Sigma), SAHA (Abcam), and Romidepsin (Selleck Chemicals) were used at a final concentration of 0.2, 5, and 5 µM, respectively. Hydroxyurea (Sigma, H8627-100G) was used at final concentration of 1 mM and phleomycin (Sigma) was used at final concentration of 500 µM.

### Transfections, siRNAs, and plasmids

Cells were transfected with siRNAs using RNAiMAX (Invitrogen) according to the manufacturer’s instructions. Cells were transfected twice with siRNAs at 0 and 24 h at a concentration of 40 nM and were analyzed 48 h after the second transfection. siRNA sequences are listed in Table 1. Cells were transfected with plasmid DNA using Lipofectamine 2000 (Invitrogen) or Xfect (Clontech) according to the manufacturer’s instructions and analyzed 24–48 h after transfection. Expression vectors for full length human CHD7 WT (pCDNA3.1-FLAG-His-CHD7-WT) and ATPase-dead (pCDNA3.1-FLAG-His-CHD7-K998R), which were a gift from Joanna Wysocka^20^, were modified by in-frame N-terminal cloning of enhanced green fluorescent protein (EGFP) or mCherry. EGFP and mCherry cDNA was amplified from pEGFP-C1 and pmCherry-C1 respectively, and cloned as an *Asc*I fragment to generate pCDNA3.1-FLAG-His-EGFP-CHD7-WT or pCDNA3.1-FLAG-His-mCherry-CHD7-WT and pCDNA3.1-FLAG-His-EGFP-CHD7-K998R or pCDNA3.1-FLAG-His-mCherry-CHD7-K998R (Table 2). siCHD7-1-resistant CHD7 cDNA was generated by introducing the underlined mutations: GAAAATAAGGATAGCGAAAAG by polymerase chain reaction (PCR) and cloned as *Not*I*/Xma*I fragment into pCDNA3.1-FLAG-His-mCherry-CHD7-WT and pCDNA3.1-FLAG-His-mCherry-CHD7-K998R (Table 2). macroH2A1.1 cDNA was obtained from pmCherry-macroH2A1.1^25^ and cloned as an *Xma*I/*Bgl*II fragment into piRFP670-C3 to generate piRFP670-mH2A1.1. All other plasmids were described previously: pPAGFP-H2A^24^, pmEGFP-ALC1, pPTagRFP-H2B^23^, piRFP670-ALC1 wildtype (WT), piRFP670-ALC1 ATPase-dead (E175Q), pLacI-GFP trap^22^, pYFP-mH2A1.1 macro domain^57^, and pPARP1-mCherry^58^.

**Table 1:** List of siRNAs.

Target	Sequence (5′–3′)
53BP1	UAUUACCGUCUCCUCGUUC
BRCA1	AGAUAGUUCUACCAGUAAA
BRCA2	GAAGAAUGCAGGUUUAAUA
BRG1	SMARTpool: ON-TARGETplus (Dharmacon)
CHD7	GGGAUUAGUUAACAAUACA
esiRNA human CHD7	EHU051311-20UG (Sigma)
CHD7-1	GAACAAAGACUCUGAGAAA
CHD7-3	CGAAAGAACCCAAGGAGAA
CHD7-4	GGAACAAGCCGAAGGCAAA
CHD7-42	GGGAUUAGUUAACAAUACA
CHD4	GAGCGGCAGUUCUUUGUGA
Control	SilencerSelect Negative Control siRNA (Ambion, ThermoFisher)
DNA-PKcs	CUUUAUGGUGGCCAUGGAG
HDAC1	CAGCGACUGUUUGAGAACC
HDAC2	GCGGAUAGCUUGUGAUGAA
Ku80	CAAGGAUGAGAUUGCUUUAGU
LIG3	GAACUGUGCCUAUUCCGAA
Luciferase	CGUACGCGGAAUACUUCGA
PARP1	CCAUCGAUGUCAACUAUGA
PARP2	CCAUUGGACCAACACUAUA
PARP3	CAGACCUACUUAGAACAGA
POLQ	CCGCUUUUGGAGUCAGUAATT
p53	GACUCCAGYGGUAAUCUAC
RNF8	GGGUCUAUUCCAUUCAUCA
RNF168	GUGGAACUGUGGACGAUAA
XRCC4	AUAUGUUGGUGAACUGAGA

**Table 2:** List of primers.

Name	Sequence (5′–3′)
EGFP_AscI FW	TTAATTGGCGCGCCTTGTGAGCAAGGGCGAGGAG
EGFP_AscI RV	GATACTGGCGCGCCTGCTTGTACAGCTCGTCCATG
CHD7-1_siRNAres_NotI FW	GGTTTTGTTCCTGAGTCGATGTTTGACCGCCTTC
CHD7-1_siRNAres_NotI RV	CTGTGCTCTTTTCGCTATCCTTATTTTCGTCTTC
CHD7-1_siRNAres_XmaI FW	GAAGACGAAAATAAGGATAGCGAAAAGAGCACAG
CHD7-1_siRNAres_XmaI RV	GAATTCGAAGCTTGAGCTCTAGCATTTAGGTGAC
CMV1 FW	TGGCCCGCCTGGCATTATGCC
CD4int RV	GCTGCCCCAGAATCTTCCTCT
M13 FW	GTAAAACGACGGCCAGT
M13 RV	CAGGAAACAGCTATGAC

### Generation of DSBs by IR

IR was delivered to cells by an YXlon X-ray generator machine (200 KV, 4 mA, dose rate 1 Gy/minute) or a Faxitron Cabinet X-ray System Model RX-650 (130 kVp, dose rate 1.85 Gy/min).

### 365 nm UV-A laser micro-irradiation

Cells were grown on 18 mm coverslips and sensitized with 10 µM 5′-bromo-2-deoxyuridine (BrdU) for 24 h as described^24^. For micro-irradiation, the cells were placed in a Chamlide TC-A live-cell imaging chamber that was mounted on the stage of a Leica DM IRBE widefield microscope stand (Leica) integrated with a pulsed nitrogen laser (Micropoint Ablation Laser System; Andor). The pulsed nitrogen laser (16 Hz, 364 nm) was directly coupled to the epifluorescence path of the microscope and focused through a Leica 40× HCX PLAN APO 1.25–0.75 oil-immersion objective. The growth medium was replaced by CO_2_-independent Leibovitz’s L15 medium supplemented with 10% fetal calf serum (FCS) and cells were kept at 37 °C. The laser output power was set to 72–80 to generate strictly localized sub-nuclear DNA damage. Cells were micro-irradiated (two iterations per pixel) within 5 min using Andor IQ software (Andor). Following micro-irradiation, cells were incubated for the indicated time points at 37 °C in Leibovitz’s L15 and subsequently fixed with 4% formaldehyde before immunostaining. Images of fixed samples were acquired on a Zeiss AxioImager M2 or D2 widefield fluorescence microscope equipped with 40×, 63×, and 100× PLAN APO (1.4 NA) oil-immersion objectives (Zeiss), an HXP 120 metal–halide lamp used for excitation and the following filters: DAPI (excitation filter: 350/50 nm, dichroic mirror: 400 nm, emission filter: 460/50 nm), GFP/Alexa 488 (excitation filter: 470/40 nm, dichroic mirror: 495 nm, emission filter: 525/50 nm), mCherry (excitation filter: 560/40 nm, dichroic mirror: 585 nm, emission filter: 630/75 nm), Alexa 555 (excitation filter: 545/25 nm, dichroic mirror: 565 nm, emission filter: 605/70 nm), and Alexa 647 (excitation filter: 640/30 nm, dichroic mirror: 660 nm, emission filter: 690/50 nm). Images were recorded using ZEN 2012 software and analyzed in Image J as described previously^24^. Briefly, the average pixel intensity of laser tracks was measured within the locally irradiated area (Idamage), in the nucleoplasm outside the locally irradiated area (Inucleoplasm), and in a region not containing cells in the same field of view (Ibackground). The level of protein accumulation relative to the protein level in the nucleoplasm was calculated as follows: ((*I*_damage_ − *I*_background_)/(*I*_nucleoplasm_ − *I*_background_) − 1).

### 405 nm laser micro-irradiation

Laser micro-irradiation and local photoactivation at 405 nm was performed using a single-point scanning head (iLas2 from Roper Scientific) on a Nikon Ti-E inverted microscope equipped with a spinning-disk scan head CSU-X1 from Yokogawa at a rotation speed of 5000 rpm, a Plan APO 60×/1.4 N.A oil-immersion objective lens and a sCMOS ORCA Flash 4.0 camera. U2OS cells stably expressing H2B-PAGFP were seeded on LabTek II chambered coverglass (Thermo Scientific) and sensitized with fresh medium containing 0.3 μg/mL Hoechst 33342 for 1 h at 37 °C. Prior to imaging, the medium was replaced with CO_2_-independent phenol red-free Leibovitz’s L15 medium (Life Technologies) supplemented with 20% FCS. Nuclei were irradiated with 405 nm light with a 16 μm line through the nucleus and images were collected every 4 s. Chromatin relaxation quantification has been previously described^23^. Briefly, the change in the width of the photoactivated line gives a readout of chromatin relaxation. Using a custom Matlab routine, the width of the photoactivated line is automatically segmented and measured at different time points after irradiation. For chromatin relaxation in the presence of HDAC inhibitors were added 5 min prior to irradiation. For chromatin recondensation in the presence of HDAC inhibitors, nuclei were irradiated and imaged for 3 min until chromatin had fully relaxed. Cells were then incubated with HDAC inhibitors for 3 min before imaging was resumed. Hypotonic treatment was administered to samples after chromatin had fully relaxed as previously described^47^. Z-stacks (1 µm steps) of irradiated nuclei were collected every minute for 35 min. The width of the irradiated line was measured using the Matlab routine applied to the maximum intensity projections of the Z stacks. The width of the photoactivated line was normalized to the first image recorded after addition of HDAC inhibitors or hypotonic treatment.

### Multiphoton laser micro-irradiation

Cells grown were grown on 18 mm coverslips. For micro-irradiation, cells were placed in a Chamlide CMB magnetic chamber and the growth medium was replaced by CO_2_-independent Leibovitz’s L15 medium supplemented with 10% FCS and antibiotics. Laser micro-irradiation was performed on a Leica SP5 confocal microscope equipped with an environmental chamber set to 37 °C. DNA damage-containing tracks (1.5 μm width) were generated with a Mira mode locked titanium–sapphire (Ti:Sapphire) laser (*l* = 800 nm, pulse length = 200 fs, repetition rate = 76 MHz, output power = 80 mW) using a UV-transmitting 63 × 1.4 NA oil-immersion objective (HCX PL APO; Leica). Confocal images were recorded before and after laser irradiation at 5 or 30 s time intervals over a period of 3–20 min. PAGFP-H2A was photoactivated using the same laser and settings as those used to inflict localized DNA damage. Images after multiphoton micro-irradiation of living cells were recorded using LAS-AF software (Leica) and analyzed with Image J as described previously^24^. The average pixel intensity of laser tracks was measured within the locally irradiated area (Idamage), in the nucleoplasm outside the locally irradiated area (Inucleoplasm) and in a region not containing cells in the same field of view (Ibackground). The level of protein accumulation relative to the protein level in the nucleoplasm was calculated as follows: ((*I*_damage_ − *I*_background_)/(*I*_nucleoplasm_ − *I*_background_) − 1). The width of the photoactivated line of PAGFP-H2A was measured at 5 s and 120 s or 20 min using ImageJ software. The absolute track width was calculated as a difference in the line width between 120 s or 20 min and 5 s.

### Immunofluorescence analysis

Cells were either directly fixed with 4% formaldehyde in phosphate-buffered saline (PBS) for 15 min at room temperature (RT), or pre-extracted with 0.25% Triton-X100 (Serva) in cytoskeletal buffer (10 mM Hepes-KOH, 300 mM Sucrose, 100 mM NaCl, 3 mM MgCl_2_, pH 7.4) on ice for 2 min prior to fixation. Alternatively, cells were fixed, post-extracted with 0.5% Triton-X100 (Serva) in PBS and treated with 100 mM glycine in PBS for 10 min to block unreacted aldehyde groups. Cells were then rinsed with PBS and equilibrated in wash buffer (PBS containing 0.5% bovine serum albumin and 0.05% Tween 20). Antibody incubation steps and washes were in wash buffer. Primary antibodies were incubated for 1–2 h at RT. Detection was done using goat anti-mouse or goat anti-rabbit Ig coupled to Alexa 488, 555, or 647 (1:1500; Invitrogen Molecular probes). All antibodies are listed in Table 3. Samples were incubated with 0.1 μg/mL 4′,6-Diamidino-2-Phenylindole Dihydrochloride (DAPI) and mounted in Polymount.

**Table 3:** List of primary antibodies.

Protein	Host	Company	IF	WB
53BP1	Rabbit	Novus Biologicals (NB100-304)	1:1000	1:2000
53BP1	Mouse	BD Biosciences (612522)		1:1000
ATM	Rabbit	Cell Signaling (clone D2E2)		1:1000
pATM (S1981)	Mouse	Cell Signaling (4526)		1:1000
BRCA1	Mouse	Santa Cruz (sc-6954)	1:100	1:500
BRG1	Mouse	Santa Cruz (clone G7, sc-17796)		1:1000
CHD7	Rabbit	Bethyl Laboratories (A301-223A)		1:1000
CHD7	Rabbit	Novus Biologicals (NBP1-77393)	1:200	
CHD4	Rabbit	Active Motif (39289)		1:1000
CHK1	Mouse	Santa Cruz (clone G-4, sc-8408)		1:1000
pCHK1 (S345)	Rabbit	Cell Signaling (clone 133D3)		1:1000
DNA-PKcs	Mouse	Abcam (clone 18-2)		1:1000
pDNA-PKcs (S2056)	Rabbit	Abcam (ab18192)		1:1000
FK2	Mouse	Enzo Lifesciences	1:1000	
Geminin	Rabbit	Proteintech (10802-1-AP)	1:400	
GFP	Mouse	Roche (11814460001)		1:2500
H4panAc	Rabbit	Millipore (06-866)	1:2000	
HDAC1	Rabbit	Abcam (ab7028)	1:100	
HDAC1	Rabbit	Imgenex (IMG337)		1:1000
HDAC2	Rabbit	Santa Cruz (sc-7899)		1:1000
Ku80	Rabbit	Santa Cruz (H-300, sc-9034)		1:2000
LIG3	Rabbit	Abcam (ab96576)		1:1000
LIG4	Rabbit	Abcam (ab193353)	1:100	
MDC1	Rabbit	Abcam (ab11171-50)	1:1000	
p53	Mouse	Santa Cruz (clone DO-1)		1:1000
PAR	Rabbit	Enzo Lifesciences	1:1000	1:2000
PARP1	Rabbit	Cell Signaling (9542)		1:2000
PCNA	Mouse	Santa Cruz (PC10, sc-56)	1:500	1:2000
RAD51	Rabbit	Santa Cruz (sc-8349)		1:1000
RAD51	Mouse	GeneTex (clone 14B4)	1:200	
RNF8	Mouse	Santa Cruz (B-2)		1:100
RNF168	Rabbit	Millipore (ABE367)		1:500
RPA32	Mouse	Abcam (ab2175)		1:1000
pRPA32 (S4/S8)	Rabbit	Bethyl Laboratories (A300-245A)		1:1000
α-Tubulin	Mouse	Sigma (cloneDM1A, T6199)		1:5000
XRCC4	Rabbit	Gift from D. van Gent	1:500	
XRCC4	Mouse	SAB (40455)		1:1000
γH2AX	Mouse	Millipore (clone JBW301, 05-636)	1:2000	

### Monitoring nascent transcription at DNA damage sites

Transcription at sites of DNA damage was measured by labeling nascent RNA with 1 mM 5-5-ethynyl uridine (5-EU, Invitrogen), which was added 5 min after UV-A laser micro-irradiation. 5-EU incorporation was determined 1 h later by using a Click-iT RNA imaging kit (Invitrogen) as described previously^46^.

### DSB-transcription reporter assays

U2OS 2-6-3 cells stably expressing ER-mCherry-LacR-FokI-DD^21^ were treated for 3 h with 1 μM Shield-1 (Clontech Laboratories UK Ltd.) and 1 μM 4-hydroxytamoxifen (4-OHT, Sigma-Aldrich) to induce DSBs and subsequently for 3 h with 1 μg/mL doxycycline (Sigma-Aldrich) to induce transcription of the reporter gene. The number of transcription-positive cells was counted from a total of 150 cells per sample in each independent repeat as described previously^59^.

### Pulldown and co-immunoprecipitation assays

U2OS cells transiently expressing GFP-NLS, GFP–CHD7 WT, or GFP–CHD7 ATPase-dead (K998R) were used for pulldown assays, while untransfected U2OS cells were used for co-immunoprecipitation assays. Cells were lysed in EBC buffer (50 mM Tris, pH 7.5, 150 mM NaCl, 0.5% NP-40, 2.5 mM MgCl_2_, protease inhibitor cocktail tablets) with 500 units benzonase. Samples were incubated for 90 min at 4 °C under constant mixing. Totally, 50 μL Input sample was collected in a separate tube and mixed with 2× Laemmli buffer. The cleared lysates were subjected to GFP pulldown with GFP-Trap beads (Chromotek) or immunoprecipitation using a specific antibody (or corresponding IgG control) that was conjugated to Protein A-coupled agarose beads (Millipore 16–157). The beads were then washed six times with EBC buffer and boiled in 2× Laemmli buffer along with the input samples. Samples were subjected to western blot analysis.

### Western blot analysis

Cells were lysed in 2× Laemmli buffer and proteins were separated by sodium dodecyl sulfate polyacrylamide gel electrophoresis using 4–12% pre-cast polyacrylamide gels (BioRad or Invitrogen) and 20× MOPS running buffer (Invitrogen). Next, proteins were transferred onto nitrocellulose membranes (Millipore). Protein expression was analyzed by immunoblotting with the indicated primary antibodies (Table 3) and secondary CF680 goat anti-rabbit or CF770 goat anti-mouse Ig antibodies (1:5000, Biotium). Membranes were scanned and analyzed using a Licor Odyssey scanner (LI-COR Biosciences).

### Sample preparation and MS analysis

For SILAC labeling, U2OS cells were cultured for 14 days in media containing “heavy” (H) “light” (L) labeled forms of the amino acids arginine and lysine, respectively. SILAC-labeled cells were transiently transfected with a GFP–CHD7 (H) or GFP-NLS expression vector (L) and equal amounts of H- and L-labeled cells were lysed in EBC buffer as described above. GFP–CHD7 (H) and GFP-NLS (L) lysates were subjected to immunoprecipitation using GFP-Trap beads as described above. The beads were subsequently washed 2 times with EBC buffer and 2 times with 50 mM (NH_4_)_2_CO_3_ followed by overnight digestion using 2.5 μg trypsin at 37 °C under constant shaking. Peptides of the H and L precipitates were mixed in a 1:1 ratio and desalted using a Sep-Pak tC18 cartridge by washing with 0.1% acetic acid. Finally, peptides were eluted with 0.1% acetic acid/60% acetonitrile and lyophilized.

MS was performed as described previously^60^. Sample were analyzed on a Q-Exactive Orbitrap mass spectrometer (Thermo Scientific) coupled to an EASY-nanoLC 1000 system (Proxeon). Digested peptides were separated using a 13 cm fused silica capillary (ID: 75 μm, OD: 375 μm, Polymicro Technologies, California, USA) in-house packed with 1.8 μm C18 beads (ReprospherDE, Pur, Dr. Maisch). Peptides were separated by liquid chromatography using a gradient from 2 to 95% acetonitrile with 0.1% formic acid at a flow rate of 200 nL/min for 2 h. The mass spectrometer was operated in positive-ion mode at 2.2 kV with the capillary heated to 200 °C. Data-dependent acquisition mode was used to automatically switch between full scan MS and MS/MS scans, employing a top ten method. Full scan MS spectra were obtained with a resolution of 70,000, a target value of 3 × 10^6^ and a scan range from 400 to 2000 m/z. Higher-collisional dissociation tandem mass spectra (MS/MS) were recorded with a resolution of 17,500, a target value of 1 × 10^5^ and a normalized collision energy of 25%. The precursor ion masses selected for MS/MS analysis were subsequently dynamically excluded from MS/MS analysis for 60 s. Precursor ions with a charge state of 1 and greater than 6 were excluded from triggering MS/MS events. The raw MS file was analyzed with the MaxQuant software suite (version 1.5.5.1; Max Planck Institute of Biochemistry). The data have been deposited to the ProteomeXchange Consortium via the PRIDE partner repository (https://www.ebi.ac.uk/pride/archive/login) with the data set identifier PXD014339.

### DR-GFP, EJ5-GFP, and EJ2-GFP reporter assays

U2OS cells containing either a stably integrated copy of the DR-GFP, EJ5-GFP, or EJ2-GFP reporter were used to measure the repair of I-*Sce*I-induced DSBs by HR, total NHEJ or altNHEJ^27,55^. Briefly, DR-GFP U2OS cells knockout for CHD7 or EJ5-GFP and EJ2-GFP U2OS cells treated with siRNA for 48 h were co-transfected with an mCherry expression vector and the I-*Sce*I expression vector pCBASce^55^. Forty-eight hour later the percentage of GFP-positive cells among the mCherry-positive cells was determined by fluorescence-activated cell sorting (FACS) on a BD LSRII flow cytometer (BD Bioscience) using FACSDiva software version 5.0.3 (Supplementary Fig. 10g). Quantifications were performed with FACSDiva^™^ (BD Biosciences).

### Random plasmid integration assay

U2OS cells were seeded (day 1) and transfected with siRNAs the following day (day 2). Later at day 2, the cells were transfected with 2 μg gel-purified *Bam*HI/*Eco*RI-linearized pEGFP-C1 plasmid. The cells were subsequently transfected twice with siRNAs at 24 and 36 h after the first transfection (day 3 and day 4, respectively). On day 5, cells were collected, counted, seeded, and grown in medium without or with 0.5 mg/mL G418. The transfection efficiency was determined on the same day by FACS analysis using GFP fluorescence as a measure (Supplementary Fig. 10g). The cells were incubated at 37 °C to allow colony formation and medium was refreshed on day 8 and 12. On day 15, the cells were washed with 0.9% NaCl and stained with methylene blue (2.5 g/L in 5% ethanol, Sigma-Aldrich). Colonies of more than 50 cells were scored. Random plasmid integration efficiency was scored as the number of G418-resistant colonies normalized by the plating efficiency, which was determined by the number of colonies formed on plates without G418 and corrected for the transfection efficiency.

### Analysis of repair junctions in the GC92 reporter

Sequence analysis of repair junctions in the GC92 reporter was performed as described^28^. Briefly, GC92 fibroblasts were first transfected with siRNAs and 48 h later with the I-*Sce*I expression vector pCBASce. Forty-eight hour later, genomic DNA was extracted using phenol:chloroform:isoamyl alcohol (25:24:1 v/v, Invitrogen). PCR was performed on the genomic DNA using the CMV1 and CD4int primers (Table 2) to amplify repair junctions. PCR products were cloned into pGEM-T easy vector (Promega). Colony PCR was performed using M13 primers (Table 2) on individual bacterial colonies to amplify repair junctions, which were subjected to Sanger sequencing using the M13 FW primer (Table 2). Sequences were analyzed using a custom Sanger sequence analyzer as described previously^61^.

### Cell survival assays

VH10-SV40 cells were transfected with siRNAs, trypsinized, seeded at low density and exposed to IR. U2OS cells were seeded at low densities and exposed to increasing doses of olaparib. WT and CHD7 KO-2 U2OS cells were transfected with siRNAs, trypsinized and seeded at low density without DNA damage treatment. After 7 days, the cells were washed with 0.9% NaCl and stained with methylene blue (2.5 g/L in 5% ethanol, Sigma-Aldrich). Colonies of more than 20 cells were scored.

### RNA sequencing

RNA isolation was done using the miRNeasy minikit (Qiagen). The RNA 6000 Nano kit (Agilent Technologies) was used to confirm RNA integrity before the RNA was subjected to poly(A) enrichment. cDNA synthesis, library preparation and sequencing were carried out using the Ion Total RNA-Seq kit v2, the Ion PI Template OT2 200 kit v3 and the Ion Sequencing 200 kit v3, respectively, according to the manufacturer’s instructions (Invitrogen). RNA was sequenced on an Ion Proton System at a depth of approximately 20 million reads per sample, with a median read length of 90 bp. Sequence files obtained in the bam format were converted to fastq using the bam2fastq conversion utility from the bedtools package. Reads were aligned to the human genome build GRCh37—Ensembl using Tophat2 (version 2.0.10). In a second alignment step, Bowtie2 (version 2-2.10) was used in the local, very sensitive mode to align remaining un-aligned reads. HTSeq-Coumt (version 0.6.1) was used with default settings to quantify gene expression. Finally, DESeq (version 1.2.10) was used to generate a list of genes differentially expressed between CHD7-depleted and control cells. A list of DSB repair genes was obtained from the Gene Ontology (GO) functional category at http://rgd.mcw.edu/rgdweb/ontology/annot.html?accid=GO:0006302&species=Human-annot. The data have been deposited to the SRA database (https://www.ncbi.nlm.nih.gov/sra) with the following accession number: PRJNA547697.

### Cell cycle profiling

Cells were fixed in 70% ethanol, followed by DNA staining with 50 µg/mL propidium iodide in the presence of RNase A (0.1 mg/mL; Sigma). Cell acquisition and quantification was performed on a BD LSRII flow cytometer (BD Bioscience) using FACSDiva software version 5.0.3.

### Fluorescence three-hybrid assay

Florescence three-hybrid assays were performed as described^22^. Briefly, GFP-tagged proteins were tethered to a genomically integrated LacO array using a LacI-GFP trap in U2OS-2B2 cells^56^ expressing mCherry-PARP1. Cells were sensitized with Hoechst and micro-irradiated with 405 nm light to induce DNA damage. If the GFP-tagged protein of interest is able to bind PAR, PARylated mCherry-PARP1, which is generated at sites of DNA damage, will enrich at the LacO array after DNA damage induction. The mCherry-PARP1 signal intensity at the LacO array was quantified pre and 60 s post DNA damage induction. The average intensity was normalized to the average intensity of the nucleus and corrected for background signal.

### RNAi screen and quantitative image-based cytometry (QIBC)

For QIBC, cells were grown on sterile 12 mm glass coverslips, fixed in 3% formaldehyde in PBS for 15 min at RT, washed once in PBS, permeabilized for 5 min at RT in PBS supplemented with 0.2% Triton X-100 (Sigma-Aldrich), and washed twice in PBS. All primary and secondary antibodies were diluted in filtered DMEM containing 10% FBS and 0.02% Sodium Azide. Antibody incubations were performed for 1–2 h at RT. Following antibody incubations, cells were washed once with PBS and incubated for 10 min with PBS containing 4′,6-diamidino-2-phenylindole dihydrochloride (DAPI, 0.5 μg/mL) at RT to stain DNA. Following three washing steps in PBS, cells were briefly washed with distilled water and mounted on glass slides with 5 μL Mowiol-based mounting media (Mowiol 4.88; Calbiochem) in glycerol/Tris). The siRNA-based screen was performed by reverse-transfection of stable U2OS-shRNF168 cells cultured in CELLSTAR 96-well-plates (Greiner Bio-One) for 72 h at a cell density of 4500 cells per well at the time of transfection with Ambion Silencer Select siRNAs at a final concentration of 5 nM using HiPerFect (Qiagen) reagent. Automated multichannel wide-field microscopy for QIBC was performed on an Olympus ScanR Screening System equipped with an inverted motorized Olympus IX83 microscope, a motorized stage, IR-laser hardware autofocus, a fast emission filter wheel with single band emission filters, and a digital monochrome Hamamatsu ORCA-FLASH 4.0 V2 sCMOS camera (2048 × 2048 pixel, 12-bit dynamics). As described previously^14^ for each condition, image information of large cohorts of cells (typically at least 500 cells for the UPLSAPO 40× objective (NA 0.9), at least 2000 cells for the UPLSAPO 20× objective (NA 0.75), and at least 5000 cells for the UPLSAPO 10× (NA 0.4) and UPLSAPO 4× (NA 0.16) objectives) was acquired under non-saturating conditions at a single autofocus-directed z-position. Identical settings were applied to all samples within one experiment. Images were analyzed with the inbuilt Olympus ScanR Image Analysis Software Version 3.0.0, a dynamic background correction was applied, nuclei segmentation was performed using an integrated intensity-based object detection module based on the DAPI signal, and foci segmentation was performed using an integrated spot-detection module. All downstream analyses were focused on properly detected interphase nuclei containing a 2C-4C DNA content as measured by total and mean DAPI intensities. Mitotic cells with condensed chromosomes based on high mean DAPI were excluded from the quantifications. Fluorescence intensities were quantified and are depicted as arbitrary units. Color-coded scatter plots of asynchronous cell populations were generated with Spotfire data visualization software (TIBCO). Within one experiment, similar cell numbers were compared for the different conditions. Representative scatter plots and quantifications of individual experiments, typically containing several thousand cells per condition, are shown.

### Reporting summary

Further information on research design is available in the Nature Research Reporting Summary linked to this article.

## Supplementary information

Supplementary Information

Description of Additional Supplementary Files

Supplementary Data 1

Supplementary Data 2

Supplementary Data 3

Reporting Summary

## Data Availability

All data generated or analyzed during this study are included in this published article (and its Supplementary information files and Source data file). In addition, the RNA-Seq data have been deposited to the SRA database (https://www.ncbi.nlm.nih.gov/sra) with the accession number “PRJNA547697”. The mass spectrometry data have been deposited to the ProteomeXchange Consortium via the PRIDE partner repository with the data set identifier “PXD014339”. All data are available from the authors upon reasonable request. Source data are provided with this paper.

## Source data

Source Data
