# Supplementary material for: CHD7 and 53BP1 regulate distinct pathways for the re-ligation of DNA double-strand breaks
Source: Nat Commun. 2020 Nov 13;11:5775. doi: 10.1038/s41467-020-19502-5 (PMC7666215; doi:10.1038/s41467-020-19502-5)
Supplement: Supplementary file 1 — Supplementary Information [file 41467_2020_19502_MOESM1_ESM.pdf]

Supplementary Information

CHD7 and 53BP1 regulate distinct pathways for the re-ligation of DNA double-strand breaks

Magdalena B. Rother[1,†], Stefania Pellegrino[2,†], Rebecca Smith[3], Marco Gatti[2], Cornelia Meisenberg[4], Wouter W. Wiegant[1], Martijn S. Luijsterburg[1], Ralph Imhof[2], Jessica A. Downs[4], Alfred C.O. Vertegaal[5], Sébastien Huet[3,6], Matthias Altmeyer[2,*], Haico van Attikum[1,*]

This file contains:

- Supplementary Fig. 1
- Supplementary Fig. 2
- Supplementary Fig. 3
- Supplementary Fig. 4
- Supplementary Fig. 5
- Supplementary Fig. 6
- Supplementary Fig. 7
- Supplementary Fig. 8
- Supplementary Fig. 9
- Supplementary Fig. 10

Supplementary Data1 - 3 are provided as separate files.

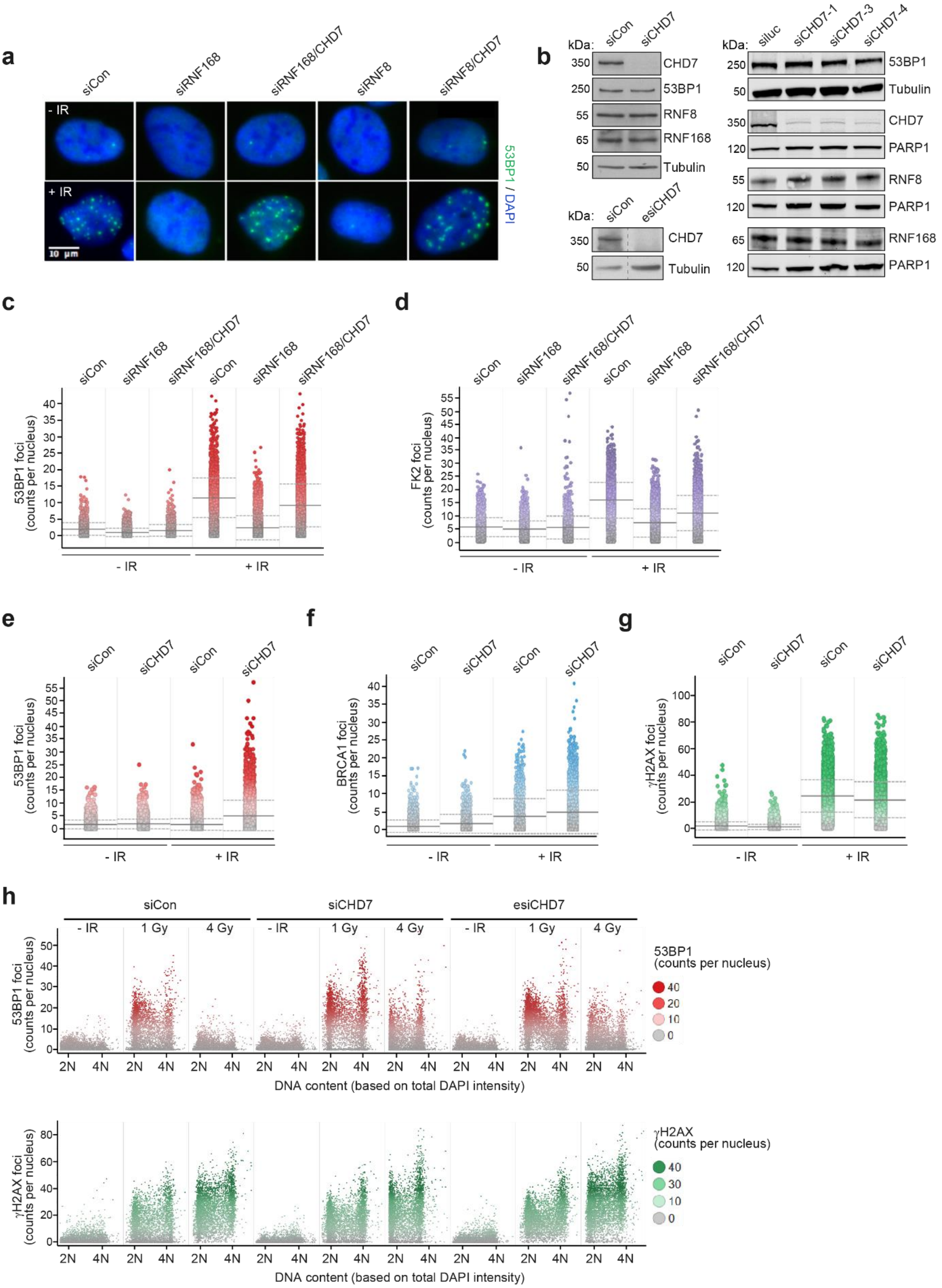

Supplementary Fig. 1 – related to Fig. 1. Validation of the targeted 53BP1 gain-of-function RNAi screen. (**a**) Representative immunofluorescent images showing 53BP1 foci formation in U2OS cells transfected with the indicated siRNAs. >1000 cells per condition were analyzed. (**b**) Western blot analysis of CHD7, 53BP1, RNF8 and RNF168 expression in CHD7-depleted cells. Tubulin and PARP1 are loading controls. Representative blots from >3

independent experiments are shown. (**c**) Quantification of single-cell QIBC analysis of >1000 cells per condition transfected with the indicated siRNAs. Cells were exposed to 0.5 Gy of IR and 53BP1 foci were quantified after 15 minutes. Mean (solid line) and standard deviation from the mean (dashed lines) is indicated. (**d**) As in c, except that cells were stained for ubiquitin conjugates with FK2 antibody. (**e**) As in c. (**f**) As in c, except for BRCA1. (**g**) As in c, except for γH2AX. (**h**) Cell cycle-resolved QIBC analysis of 53BP1 (upper panel) and γH2AX (lower panel) foci in U2OS cells transfected with the indicated siRNAs and exposed to increasing doses of IR. DNA content based on total nuclear DAPI intensity was used for cell cycle staging. Source data are provided as a Source Data file.

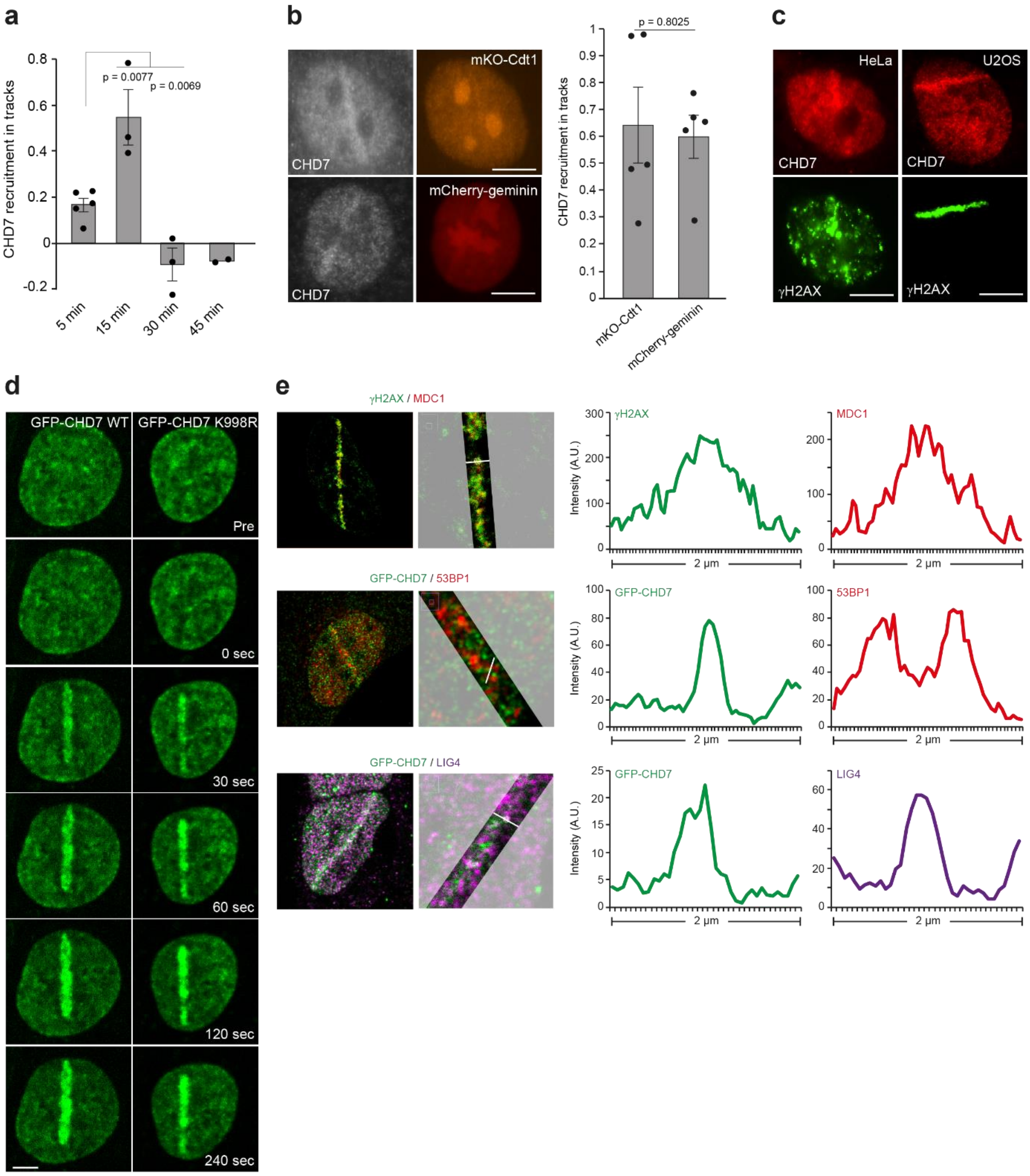

Supplementary Fig. 2 – related to Fig. 2. Kinetics of GFP-CHD7 recruitment to laser tracks. (**a**) Quantification of cells from Fig. 2c. The mean ±SEM from 2-5 independent experiments is shown. Statistical significance was calculated using the two-tailed Student's t-test. (**b**) Endogenous CHD7 recruitment to 365 nm UV-A tracks 5 minutes after damage induction in mKO-Cdt1-expressing G1-phase and mCherry-geminin-expressing S/G2-phase U2OS cells (left panel). The mean ±SEM from 5 independent experiments is shown (right panel). Statistical significance was calculated using the two-tailed Student's t-test. (**c**) Endogenous CHD7 recruitment to 365 nm UV-A tracks 15 minutes after damage induction in HeLa and U2OS cells. γH2AX is a DNA damage marker. Representative images from 1 experiment are shown. (**d**) Kinetics of GFP-CHD7 wildtype (WT) and GFP-CHD7 ATPase-dead (K998R) recruitment to 405 nm laser tracks in U2OS cells. Representative images from >2 independent experiments are shown. (**e**) For co-localization analysis at sites of pulsed UV-A micro-laser induced DNA damage, intensity distributions along 2 µm distances across micro-

foci within the laser tracks were determined from confocal images in ImageJ. Individual intensity distributions of 5-20 micro-foci per cell from 20-30 cells per condition were averaged and are displayed as means ± SD (Fig. 2e-g, 3b, 7b-c and Supplementary Fig. 7a). Here, single intensity distributions are shown as examples. Images correspond to Fig. 2. Scale bar 10 µm (b, c), 5 µm (d). Source data are provided as a Source Data file.

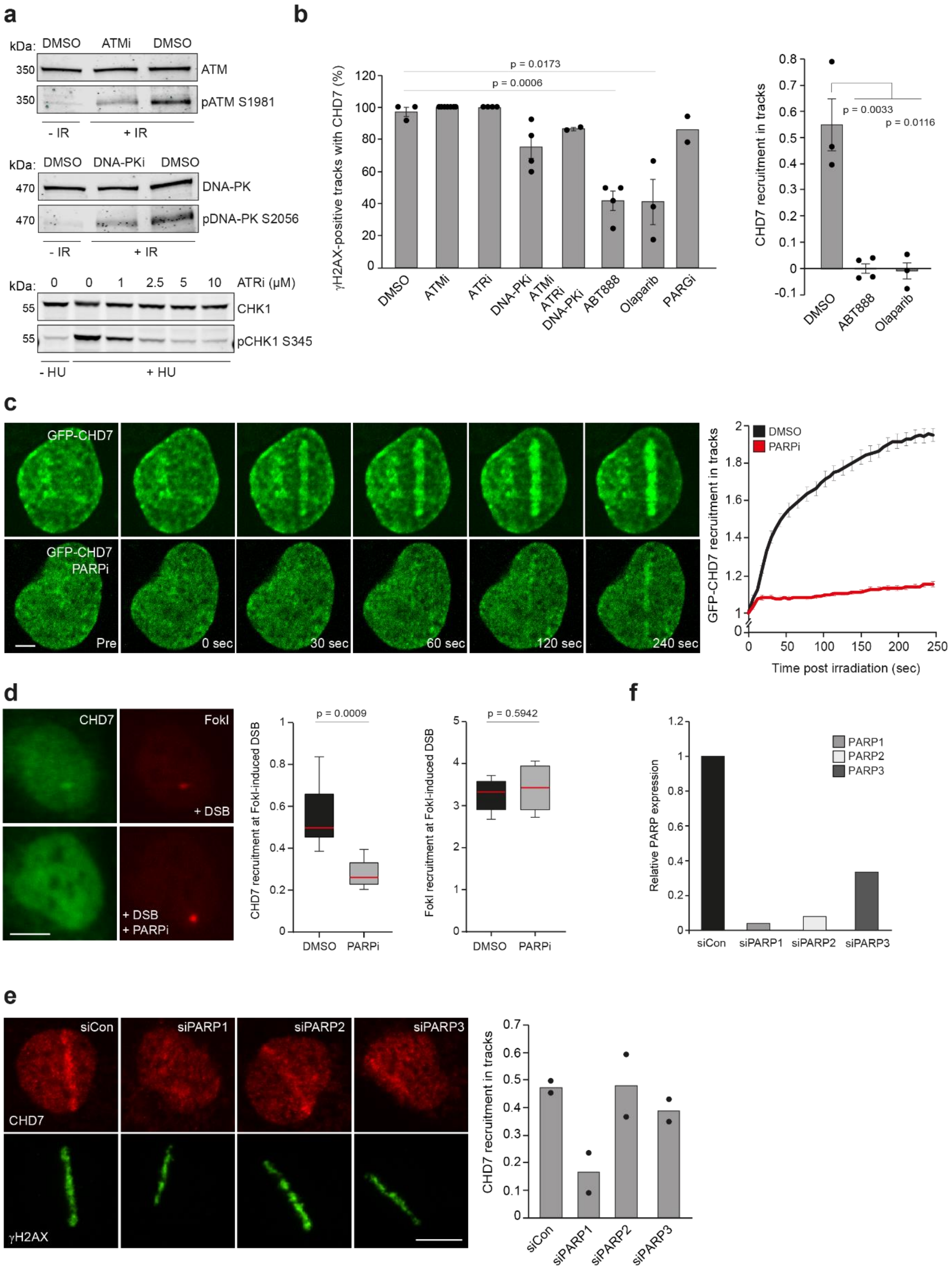

Supplementary Fig. 3 – related to Fig. 3. CHD7 recruits to DNA damage sites in a PARP1- and PAR-dependent manner. (**a**) Western blot analysis of cells from Fig. 3a. Cells were treated for 1 hour with the indicated inhibitors before DNA damage induction. DNA damage was induced with 10 Gy of IR or 1 mM hydroxyurea (HU) and cells were fixed after 1 hour or 3 hours, respectively. IR-induced ATM phosphorylation on Ser1981 and DNA-PK phosphorylation on Ser2056, and HU-induced CHK1 phosphorylation on Ser345 were

analyzed. Representative blots from 2 independent experiments are shown. (**b**) Quantification of cells from Fig. 3a. The mean ±SEM from 2-7 independent experiments is shown. Statistical significance was calculated using the two-tailed Student's t-test. (**c**) Kinetics of GFP-CHD7 recruitment to 405 nm laser tracks in U2OS cells treated for 1 hour with the PARP inhibitor olaparib prior to micro-irradiation (left panel). The mean ±SEM from 15-18 cells is shown (right panel). (**d**) As in Fig. 2d, except that PARPi (olaparib) was added 1 hour before DSB induction (left panel). Boxplots show the first, median and third quartiles from >100 cells of a representative experiment from 3 independent replicates. Statistical significance was calculated using the two-tailed Student's t-test (right panel). (**e**) CHD7 recruitment to 365 nm UV-A tracks 15 minutes after DNA damage induction in U2OS cells transfected with the indicated siRNAs. γH2AX is a DNA damage marker (left panel). The mean from 2 independent experiments is shown (right panel). (**f**) qPCR analysis of *PARP1*, *PARP2*, and *PARP3* expression in cells from e. The data were normalized to *hEif2c2* expression. Scale bar 5 µm (c), 10 µm (d, e). Source data are provided as a Source Data file.

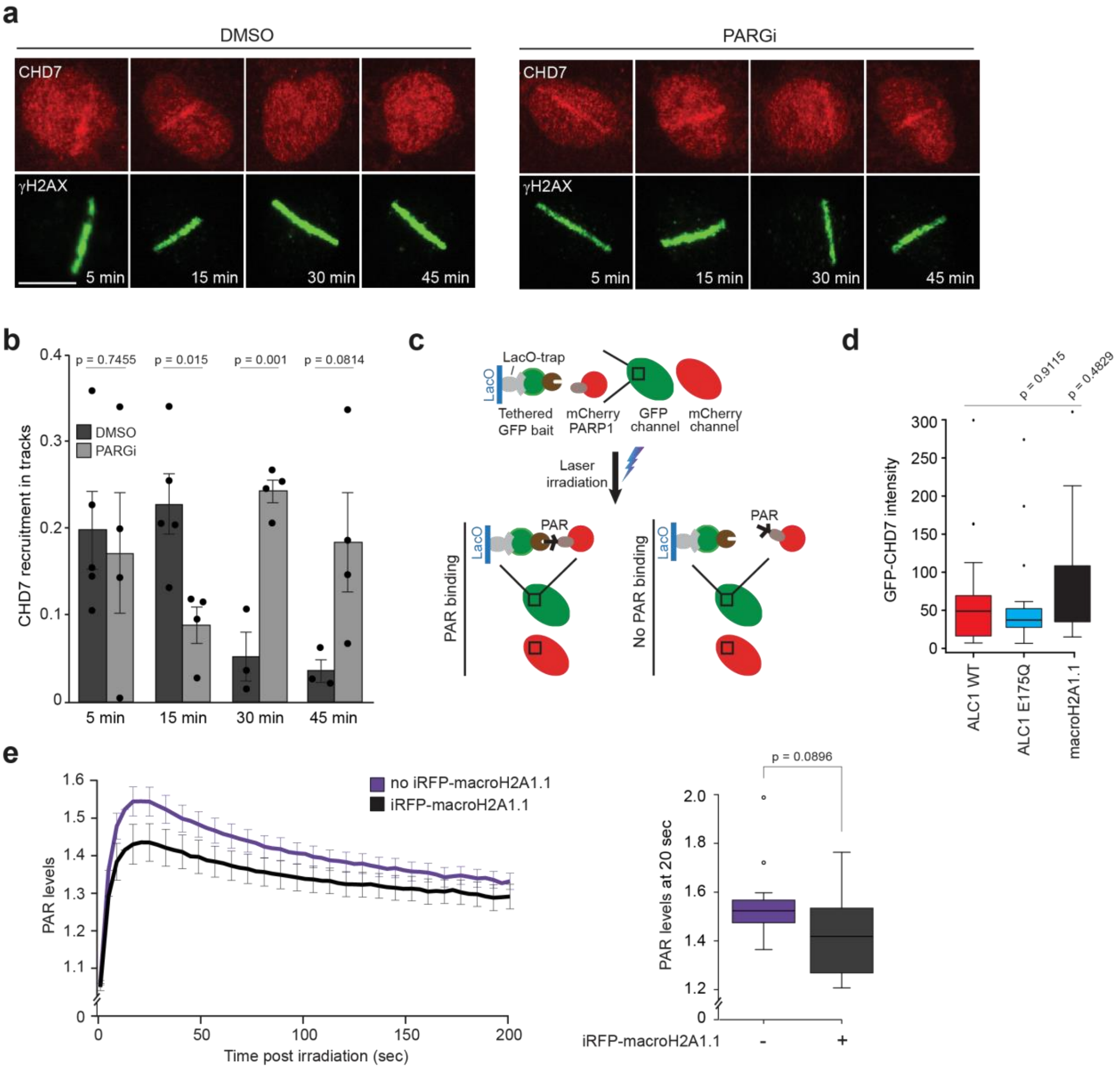

Supplementary Fig. 4 – related to Fig. 3. CHD7 is recruited to DNA damage sites in a PARP1- and PAR-dependent manner. (**a**) CHD7 recruitment to 365 nm UV-A tracks 15 minutes after DNA damage induction in U2OS cells treated for 1 hour with PARG inhibitor prior to micro-irradiation. γH2AX is a DNA damage marker. (**b**) Quantification of cells from a. The mean ±SEM from 3-5 independent experiments is shown. Statistical significance was calculated using the two-tailed Student's t-test. (**c**) Schematic of the fluorescence three-hybrid assay (F3H). Prior to laser irradiation, a GFP-tagged bait protein that is tethered to a genomically integrated LacO array though a LacI-GFP binding protein (LacO-trap) does not show enrichment of mCherry-PARP1 at the LacO array. After laser micro-irradiation mCherry-PARP1 diffuses away from the site of damage in a PARylated state. If the tethered GFP-tagged bait protein can interact with PARylated PARP1 (bottom left), mCherry-PARP1 will enrich at the LacO array. If the tethered protein of interest does not interact with the tethered bait protein (bottom right), there is no enrichment of mCherry-PARP1 at the LacO array. (**d**) Quantification of GFP-CHD7 expression in cells from Fig. 3e-f. Boxplots show the first, median and third quartiles from a representative of 3 independent replicates. Statistical significance was calculated using the two-tailed Student's t-test. (**e**) PAR levels as measured by recruitment of YFP-tagged mH2A1.1 macro domain after 405 nm laser micro-irradiation in U2OS cells expressing iRFP-macroH2A1.1 or not (left panel). Boxplot shows quantification at 20 seconds post irradiation (right panel). The first, median and third quartiles from a

representative of 3 independent replicates is shown. Statistical significance was calculated using the two-tailed Student's t-test. Scale bar 10 µm. Source data are provided as a Source Data file.

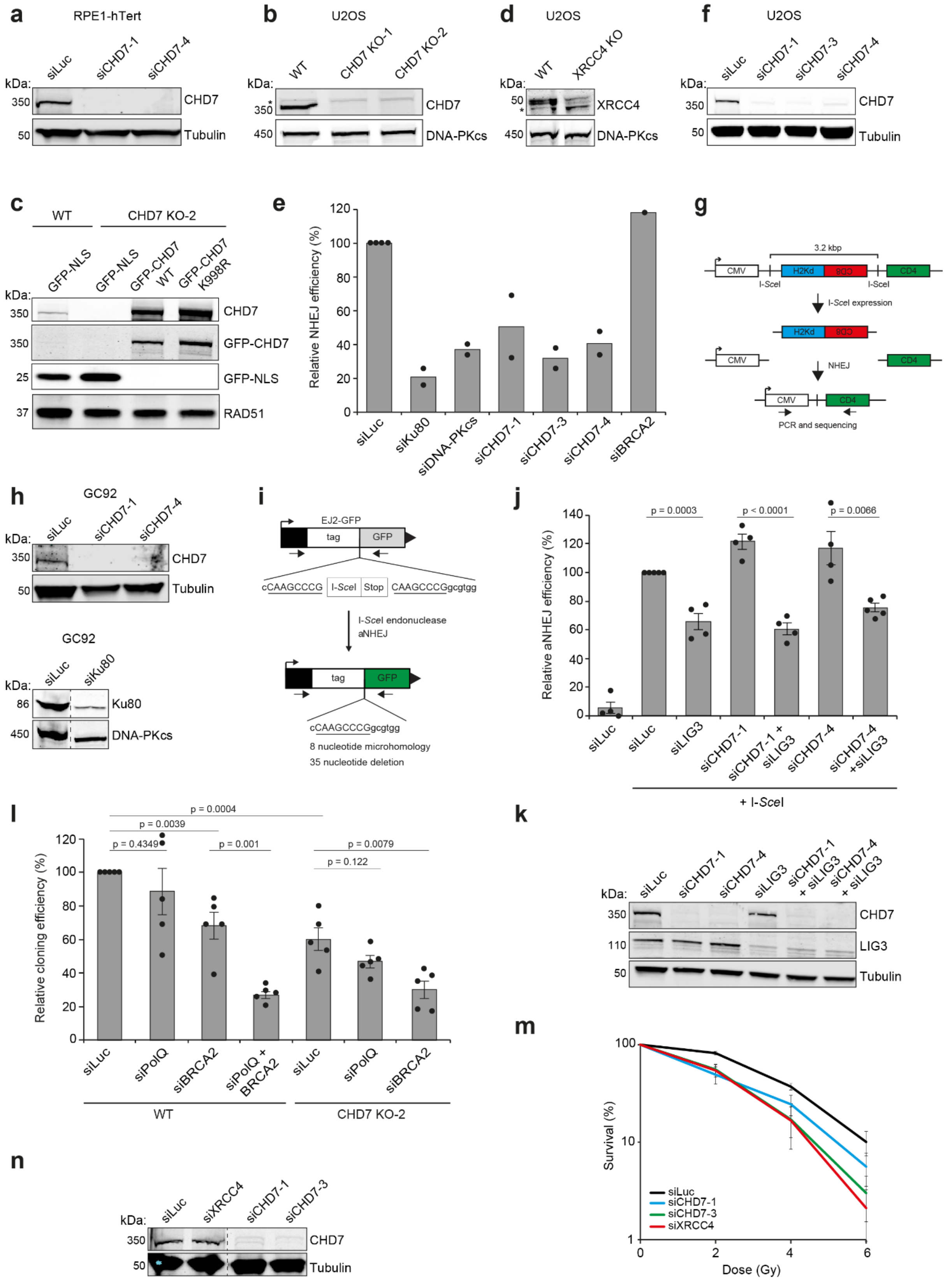

Supplementary Fig. 5 – related to Fig. 4. CHD7 promotes efficient NHEJ. (**a**) Western blot analysis of CHD7 expression in cells from Fig. 4a. Tubulin is a loading control. * Marks an unspecific band. (**b**) As in a, except in cells from Fig. 4b. DNA-PKcs is a loading control. (**c**) As in a, except that GFP-CHD7 and GFP-NLS expression was analyzed in cells from Fig. 4c. RAD51 is a loading control. (**d**) As in a, except that XRCC4 expression was analyzed in cells

from Fig. 4e. DNA-PKcs is a loading control. Representative blots from panels a-d are from >3 independent experiments. (**e**) Quantification of plasmid integration efficiencies in U2OS cells transfected with indicated siRNAs. The mean from 2 independent experiments is shown. Data were normalized to siLuc, which was set to 100%. (**f**) As in a, except in cells from e. Representative blots from >3 independent experiments are shown. (**g**) Schematic of the GC92 reporter for NHEJ. (**h**) As in a, except that CHD7 and Ku80 expression was analyzed in cells from Fig. 4f. Tubulin and DNA-PKcs are loading controls. Representative blots from 3 independent experiments are shown. (**i**) Schematic of the EJ2-GFP reporter for altNHEJ. (**j**) Quantification of EJ2-GFP-positive U2OS cells transfected with the indicated siRNAs and I-*Sce*I expression vector. I-*Sce*I transfection was corrected by co-transfection with mCherry expression vector. The mean ±SEM of 4-5 independent experiments is shown. Data were normalized to siLuc, which was set to 100%. (**k**) Western blot analysis of CHD7 and LIG3 expression in cells from j. Tubulin is a loading control. Representative blots from 3 independent experiments are shown. (**l**) Clonogenic survival of WT and CHD7 KO-2 U2OS cells transfected with the indicated siRNAs. The mean ±SEM from 5 independent experiments is shown. Data were normalized to WT, which was set to 100%. (**m**) Clonogenic survival of VH10-SV40 cells transfected with the indicated siRNAs and exposed to the indicated doses of IR. The mean ±SEM from 2 independent experiments is shown. (**n**) Western blot analysis of CHD7 expression in cells from m. Tubulin is a loading control. Representative blots from 2 independent experiments are shown. Statistical significance was calculated using the two-tailed Student's t-test (all panels). Source data are provided as a Source Data file.

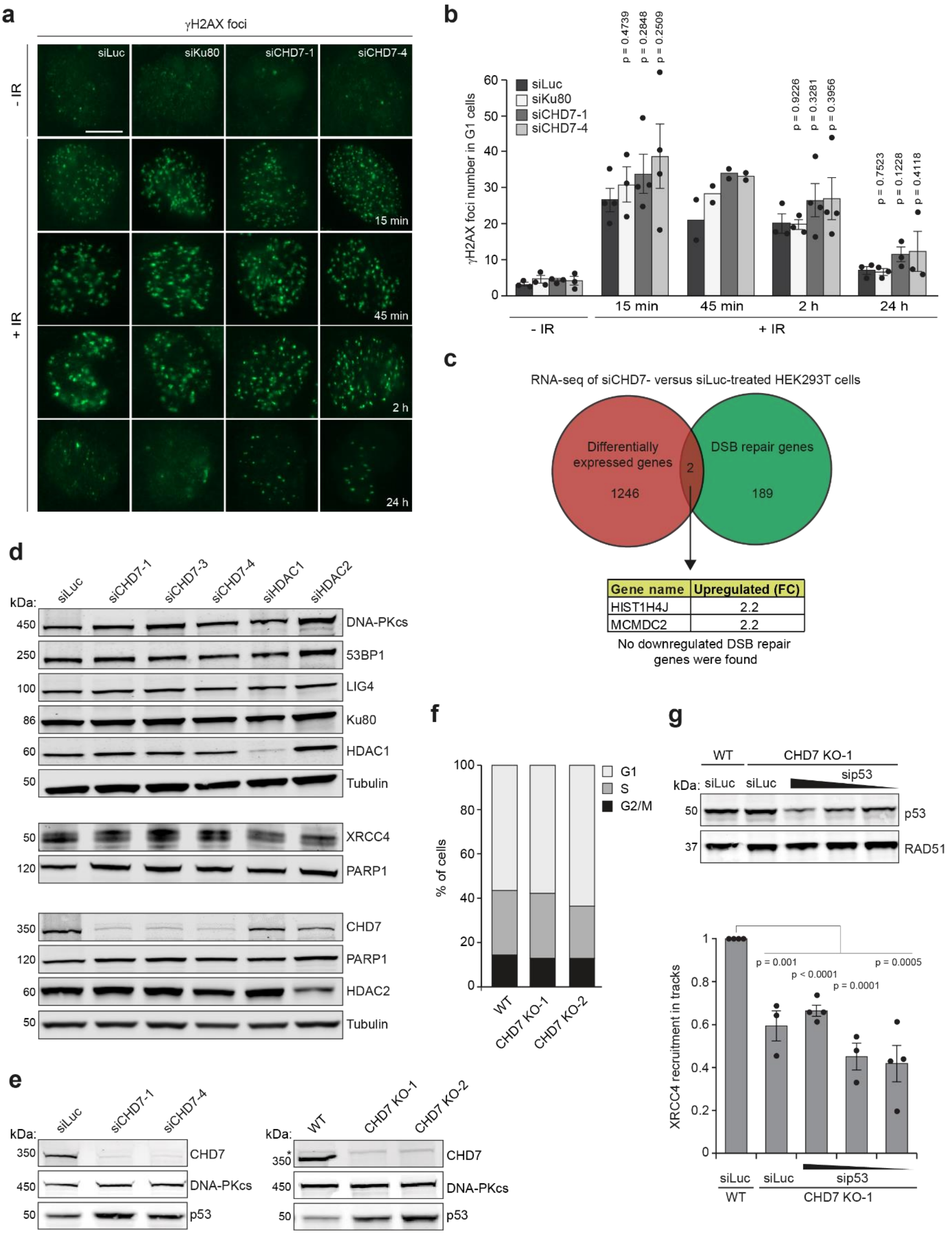

Supplementary Fig. 6 – related to Fig. 4. CHD7 promotes efficient NHEJ independently of transcription and p53 regulation. (**a**) γH2AX foci formation in U2OS cells transfected with the indicated sRNAs. Cells were exposed to 2 Gy of IR and immunostained after 15 min, 45 min, 2 hours and 24 hours. (**b**) Quantification of a. The mean ±SEM from 2-4 independent experiments. γH2AX foci were quantified in Cdt1-positive G1 phase cells. Statistical significance was calculated with the two-tailed Student's t-test. (**c**) RNA sequencing analysis of HEK293T cells transfected with siRNA against Luciferase (control) or CHD7. The data

represent the average of 3 independent replicates. Differentially expressed genes were identified using a 2-fold change cut off. A list of DSB repair genes was obtained from the Gene Ontology (GO) functional category at http://rgd.mcw.edu/rgdweb/ontology/annot.html?accid=GO:0006302&species=Humanannot. Differentially expressed DSB repair genes were examined and listed in the table S2. (**d**) Western blot analysis of DNA-PKcs, 53BP1, LIG4, Ku80, HDAC1, XRCC4, CHD7, PARP1, HDAC2 expression in U2OS cells transfected with the indicated siRNAs. Tubulin and PARP1 are loading controls. Representative blots from >3 independent experiments are shown. (**e**) Western blot analysis of CHD7 and p53 expression in U2OS cells transfected with the indicated siRNAs and in independent CHD7 knockout (KO) U2OS clones. DNA-PKcs is a loading control. * Marks an unspecific band. Representative blots from >3 independent experiments are shown. (**f**) Cell cycle profile of the indicated CHD7 KO U2OS cells. The fraction of G1-, S- and G2-phase cells was determined by propidium iodide staining and FACS analysis. (**g**) Western blot analysis of p53 expression in CHD7 KO-1 U2OS cells transfected with the indicate siRNAs. RAD51 is a loading control (upper panel). XRCC4 recruitment to 365 nm UV-A tracks 10 minutes after damage induction in CHD7 KO-1 U2OS cells transfected with siLuc and decreasing amounts of siRNA against p53. Data were normalized to wildtype (WT) cells transfected with siLuc (control), which was set to 1. The mean ±SEM from 3-4 independent experiments is shown (lower panel). Statistical significance was calculated with the two-tailed Student's t-test. Scale bar 10 µm. Source data are provided as a Source Data file.

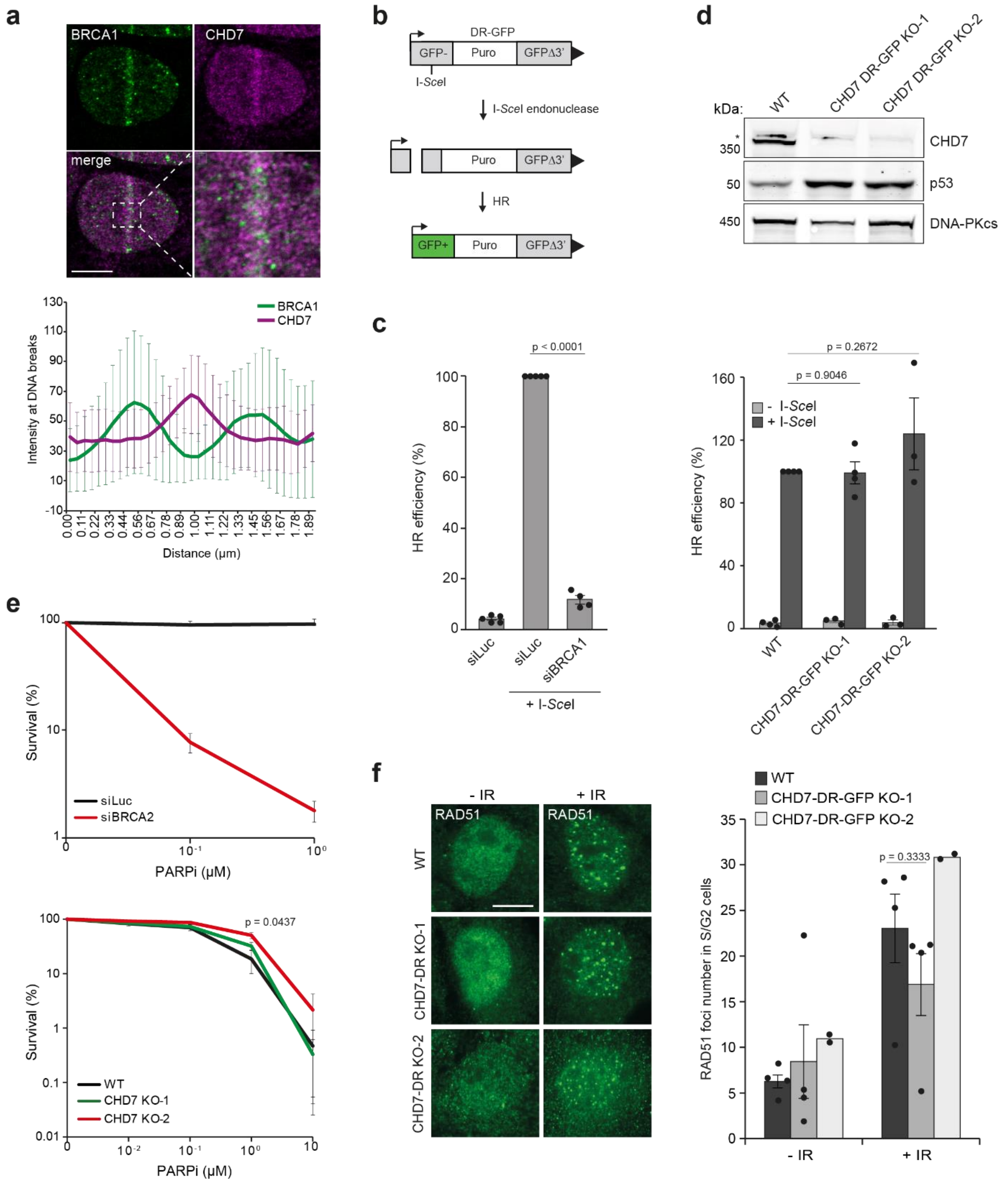

Supplementary Fig. 7 – related to Fig. 4. CHD7 is dispensable for HR. (**a**) Colocalization by confocal microscopy of CHD7 and BRCA1 at 365 nm UV-A tracks 15 minutes after DNA damage induction in U2OS cells (upper panel). Quantification of co-localized foci is shown as mean ±SD from 17 cells acquired in 3 independent experiments (lower panel). (**b**) Schematic of the DR-GFP reporter for HR. (**c**) Quantification of HR efficiencies in U2OS cells containing the DR-GFP reporter transfected with the indicated siRNAs (left panel) and 2 independent DR-GFP-containing U2OS CHD7 knockout clones (right panel). I-*Sce*I transfection was corrected by co-transfection with mCherry expression vector. The mean ±SEM from 3-6 independent experiments is shown. Statistical significance was calculated using the two-tailed Student's t-test. (**d**) Western blot analysis of CHD7 and p53 expression in the indicated DR-GFP-containing CHD7 knockout (KO) U2OS clones. DNA-PKcs is a loading control. * Marks an

unspecific band. Representative blots from >3 independent experiments are shown. (**e**) Clonogenic survival of VH10-SV40 cells transfected with the indicated siRNAs (upper panel) and of the indicated CHD7 KO U2OS clones (lower panel) following treatment with the PARP inhibitor olaparib. The mean ±SEM from 2-3 independent experiments is shown. Statistical significance was calculated using the two-tailed Student's t-test. (**f**) RAD51 foci formation in the indicated DR-GFP-containing CHD7 KO U2OS clones. Cells were exposed to 6 Gy of IR and foci were quantified after 7 hours (left panel). The mean ±SEM from >100 cells from 2-4 independent experiments is shown. Foci were quantified in immunostained, geminin-positive S/G2 phase cells (right panel). Statistical significance was calculated using the two-tailed Student's t-test. Scale bar 10 µm. Source data are provided as a Source Data file.

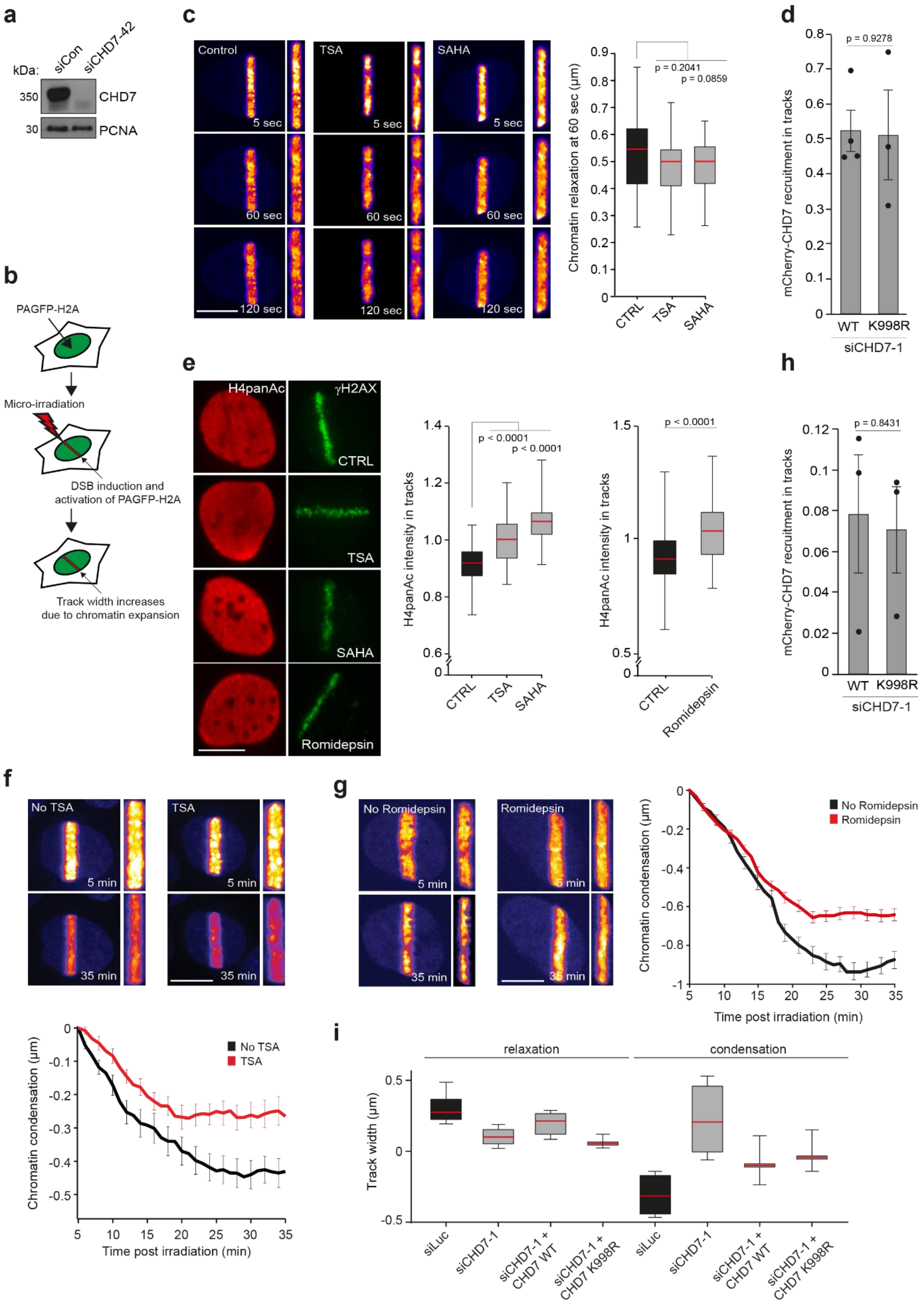

Supplementary Fig. 8 – related to Fig. 5 and 6. HDAC1/2 regulate DNA damage-induced chromatin dynamics. (**a**) Western blot analysis of CHD7 expression in cells from Fig. 5e. PCNA is a loading control. Representative blots from 3 independent experiments are shown.

(**b**) Schematic of the chromatin relaxation approach. (**c**) Chromatin relaxation in U2OS cells as measured by the thickness of the photoactivated PAGFP-H2B area in cells treated for 5 minutes with the HDAC inhibitors TSA and SAHA prior to 405 nm laser micro-irradiation (left panel). Quantification of chromatin relaxation at 60 seconds post-irradiation. Boxplot shows the first, median and third quartiles from a representative of 3 independent experiments with 24 cells per condition (right panel). Statistical significance was calculated using the two-tailed Student's t-test. (**d**) Quantification of CHD7 recruitment to 800 nm multiphoton tracks in cells from Fig. 6a. The mean ±SEM from 3-4 independent experiments is shown. Statistical significance was calculated using the two-tailed Student's t-test. (**e**) H4 de-acetylation at 365 nm UV-A tracks 15 minutes after DNA damage induction in U2OS cells treated for 5 minutes with TSA, SAHA and Romidepsin HDAC inhibitors before micro-irradiation. γH2AX is a DNA damage marker (left panel). Boxplots show the first, median and third quartiles from 41-121 cells per condition (right panel). Statistical significance was calculated using the two-tailed Student's t-test. (**f**) As in Fig. 6c, except that cells were treated with TSA. (**g**) As in Fig. 6c, except that cells were treated with Romidepsin. (**h**) As in d, except for cells from Fig. 6d. The mean ±SEM from 3 independent experiments is shown. Statistical significance was calculated using the two-tailed Student's t-test. (**i**) Kinetics of chromatin relaxation and condensation based on the data from Fig. 6a and 6d. Scale bar 10 µm. Source data are provided as a Source Data file.

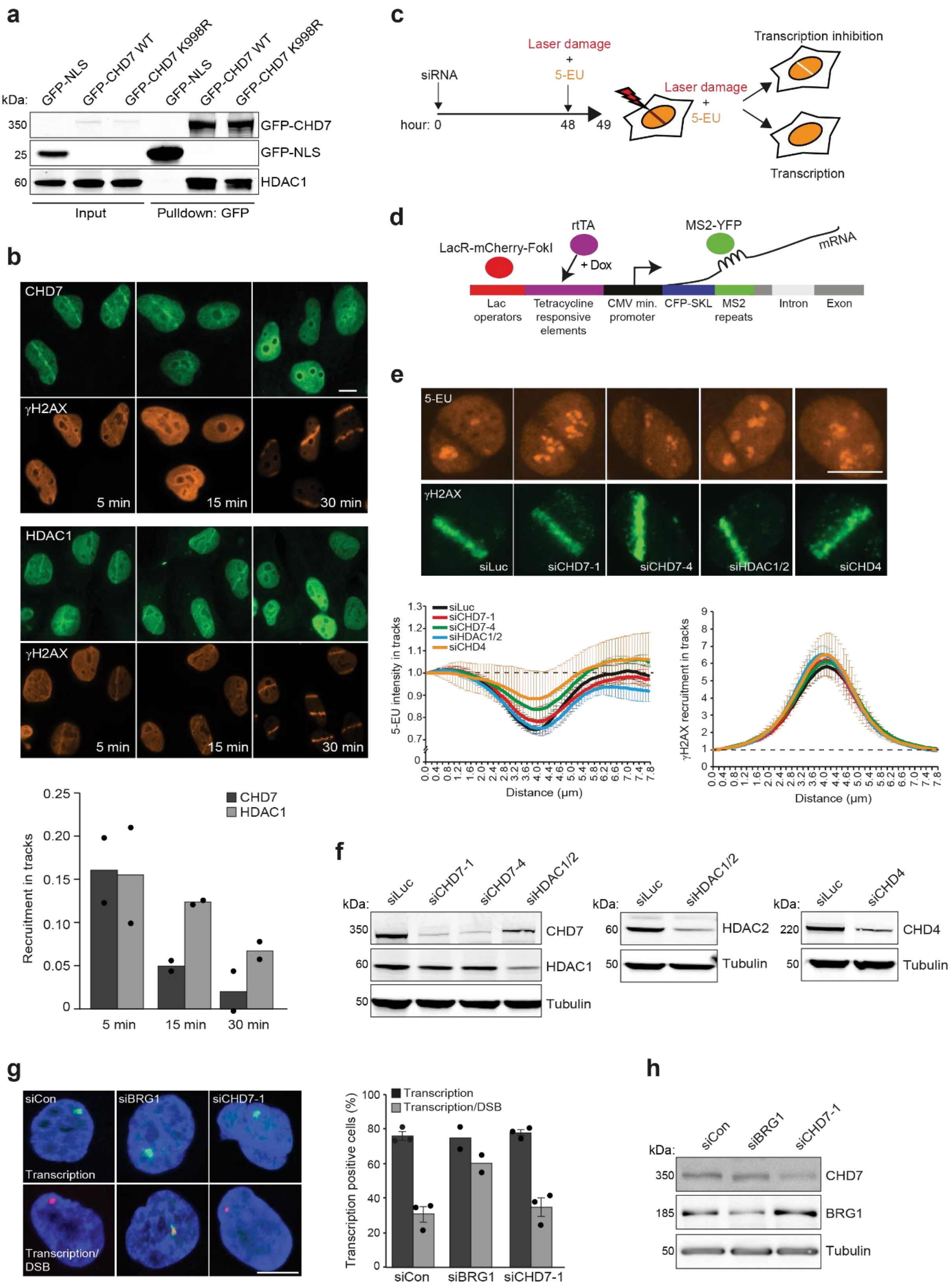

Supplementary Fig. 9 – related to Fig. 5 and 6. CHD7 is dispensable for transcription silencing around DSBs. (**a**) Pulldowns of the indicated GFP fusion proteins in U2OS cells. Blots were probed for GFP and HDAC1. Representative blots from 3 independent experiments are shown. (**b**) Kinetics of endogenous CHD7 and HDAC1 recruitment to 800 nm multiphoton tracks in U2OS cells. γH2AX is a DNA damage marker (upper panel). The mean from 2

independent experiments is shown (lower panel). (**c**) Schematic of the assay used to monitor nascent transcription by 5-ethynyl uridine (5-EU) incorporation at DNA damage sites induced by 365 nm UV-A laser micro-irradiation. (**d**) Schematic of the reporter in U2OS 2-6-3 cells, which allows us to measure doxycycline-induced transcription of a CFP-SKL-MS2 reporter at DSBs induced by the FokI endonuclease. DSBs are induced and visualized thought LacR-mCherry-FokI binding at the adjacent Lac operators, while CFP-SKL-MS2 mRNA is visualized though MS2-YFP binding. (**e**) 5-EU incorporation at UV-A tracks 1 hour after DNA damage induction in U2OS cells transfected with the indicated siRNAs. γH2AX is a DNA damage marker. siCHD4 is a positive control (upper panel). The mean ±SEM from 3 independent experiments is shown. Fluorescent intensities acquired for a cell were normalized to the maximum value measured for that cell which was set to 1. The average intensities from one experiment were normalized to the first acquired value, which was set to 1 (lower panel). (**f**) Western blot analysis of CHD7, HDAC1, HDAC2 and CHD4 expression in cells from e. Tubulin is a loading control. (**g**) YFP-MS2 transcription upon DSB induction in U2OS 2-6-3 cells transfected with indicated siRNAs. siBRG1 is a positive control (left panel). The mean ±SEM from 2-3 independent experiments is shown (right panel). (**h**) Western blot analysis of CHD7 and BRG1 expression in cells from g. Tubulin is a loading control. Scale bar 10 µm. Source data are provided as a Source Data file.

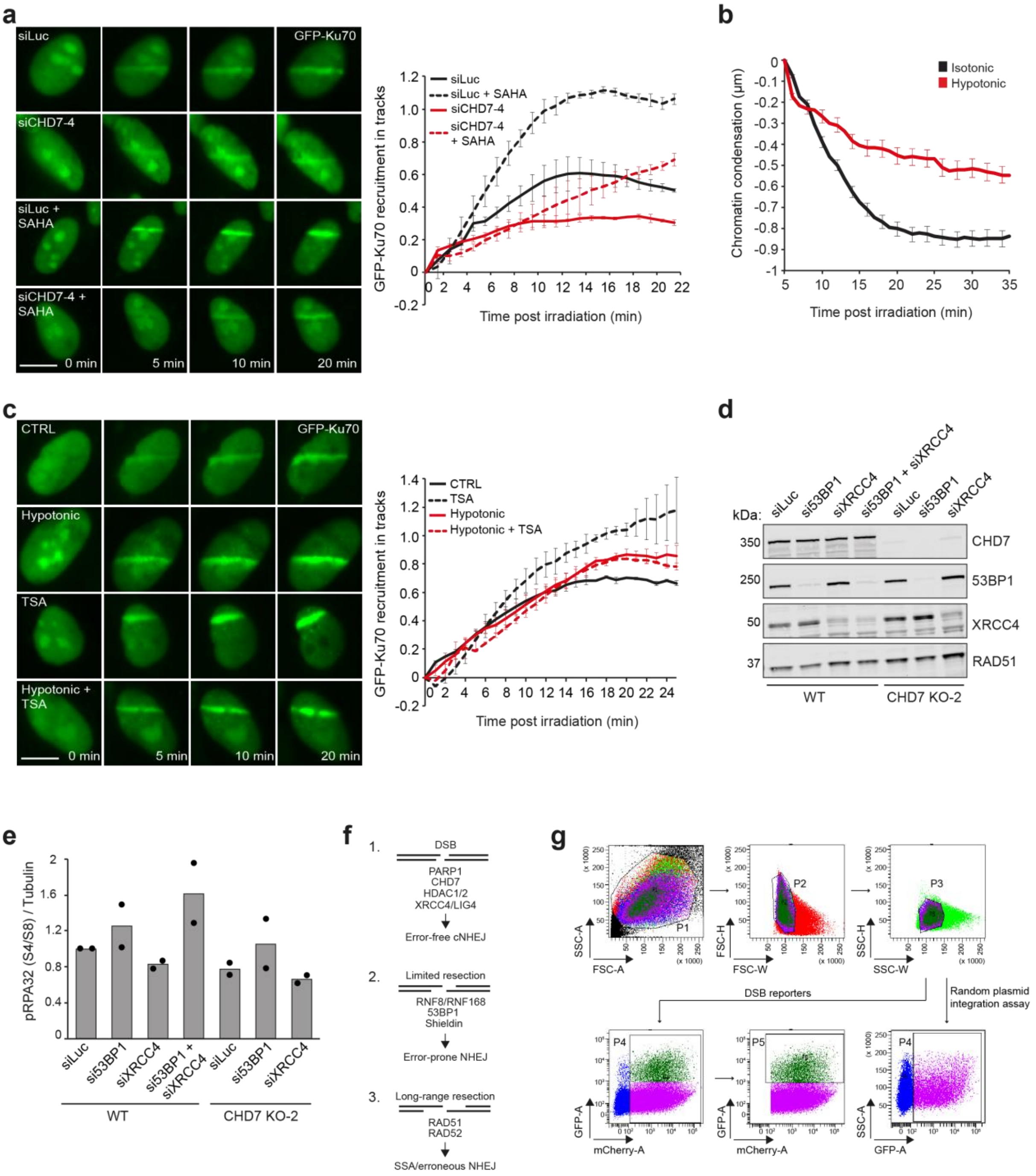

Supplementary Fig. 10 – related to Fig. 7. CHD7-HDAC1/2 and 53BP1 synergistically affect NHEJ. (**a**) GFP-Ku70 recruitment to tracks in RPE1-hTERT cells treated with the indicated siRNAs and for 5 minutes with the HDAC inhibitor SAHA before 365 nm UV-A micro-irradiation (left panel). The mean ±SEM from 2 independent experiments is shown (right panel). (**b**) Chromatin condensation in U2OS cells as measured up to 35 minutes post irradiation by the thickness of the photoactivated PAGFP-H2B area in cells in isotonic or hypotonic solutions. The mean ±SEM of 23-30 cells per condition from a representative of 3 independent experiments. (**c**) GFP-Ku70 recruitment to tracks in RPE1-hTERT cells in isotonic or hypotonic solutions and treated for 5 minutes with the HDAC inhibitor TSA before 365 nm UV-A micro-irradiation (upper panel). The mean ±SEM from 2 independent experiments is shown (lower panel). (**d**) Western blot analysis of CHD7, 53BP1 and XRCC4 expression in cells from Fig. 7e-f. RAD51 is a loading control. Representative blots from 3 independent experiments are shown. (**e**) Quantification of phosphorylated RPA32 (S4/S8)

expression in cells from Fig. 7e. Tubulin is a loading control, which was used for normalization of phosphorylated RPA32 (S4/S8) expression. The mean from 2 independent experiments is shown. Data were normalized to WT + siLuc, which was set to 1. (**f**) Organization of pathways for DSB repair by error-free NHEJ, mutagenic NHEJ and single-strand annealing (SSA) (see text for more details). (**g**) Gating strategy for flow cytometric analysis of cells for DR-GFP, EJ5-GFP and EJ2-GFP reporter assays, as well as random plasmid integration assays. Scale bar 10 µm. Source data are provided as a Source Data file.
