# Supplementary material for: CHD7 and 53BP1 regulate distinct pathways for the re-ligation of DNA double-strand breaks
Source: Nat Commun. 2020 Nov 13;11:5775. doi: 10.1038/s41467-020-19502-5 (PMC7666215; doi:10.1038/s41467-020-19502-5)
Supplement: Supplementary file 2 — Description of Additional Supplementary Files [file 41467_2020_19502_MOESM2_ESM.pdf]

Description of Additional Supplementary Files

File Name: Supplementary Data 1
Description: Average number of 53BP1 and γH2AX foci per nucleus upon siRNA-mediated depletions in the U2OS-shRNF168 background

File Name: Supplementary Data 2
Description: RNA sequencing analysis of HEK293T cells transfected with control Luciferase siRNA or siRNAs against CHD7

File Name: Supplementary Data 3
Description: Proteins identified as interactors of CHD7 by SILAC LC-MS/MS
