# Supplementary material for: CHD7 and 53BP1 regulate distinct pathways for the re-ligation of DNA double-strand breaks
Source: Nat Commun. 2020 Nov 13;11:5775. doi: 10.1038/s41467-020-19502-5 (PMC7666215; doi:10.1038/s41467-020-19502-5)
Supplement: Supplementary file 6 — Reporting Summary [file 41467_2020_19502_MOESM6_ESM.pdf]

nature research

Corresponding author(s): Haico van Attikum

Last updated by author(s): Sep 30, 2020

Reporting Summary

Nature Research wishes to improve the reproducibility of the work that we publish. This form provides structure for consistency and transparency in reporting. For further information on Nature Research policies, see our Editorial Policies and the Editorial Policy Checklist.

Statistics

For all statistical analyses, confirm that the following items are present in the figure legend, table legend, main text, or Methods section.

n/a	Confirmed	
☐	☒	The exact sample size (*n*) for each experimental group/condition, given as a discrete number and unit of measurement
☐	☒	A statement on whether measurements were taken from distinct samples or whether the same sample was measured repeatedly
☐	☒	The statistical test(s) used AND whether they are one- or two-sided<br>*Only common tests should be described solely by name; describe more complex techniques in the Methods section.*
☐	☒	A description of all covariates tested
☒	☐	A description of any assumptions or corrections, such as tests of normality and adjustment for multiple comparisons
☐	☒	A full description of the statistical parameters including central tendency (e.g. means) or other basic estimates (e.g. regression coefficient) AND variation (e.g. standard deviation) or associated estimates of uncertainty (e.g. confidence intervals)
☐	☒	For null hypothesis testing, the test statistic (e.g. *F*, *t*, *r*) with confidence intervals, effect sizes, degrees of freedom and *P* value noted<br>*Give P values as exact values whenever suitable.*
☒	☐	For Bayesian analysis, information on the choice of priors and Markov chain Monte Carlo settings
☒	☐	For hierarchical and complex designs, identification of the appropriate level for tests and full reporting of outcomes
☒	☐	Estimates of effect sizes (e.g. Cohen's *d*, Pearson's *r*), indicating how they were calculated

Our web collection on statistics for biologists contains articles on many of the points above.

Software and code

Policy information about availability of computer code

Data collection

1. MaxQuant software suite (version 1.5.5.1; Max Planck Institute of Biochemistry) (mass spectrometry)
2. Tophat2 (version 2.0.10) (RNA-Seq analysis)
3. FACSDiva software version 5.0.3 (flow cytometry)
4. Olympus ScanR Image Analysis Software Version 3.0.0 (QIBC)
5. ZEN 2012 (blue edition, version 1.1.0.0) microscopy software (wide-field microscopy)
6. LAS-AF Lite version 1.0.0 (confocal microscopy)
7. Licor Odyssey V3.0 (Western blots)
8. Image Studio Lite Ver 5.2

Data analysis

1. TIDE: Easy quantitative assessment of genome editing by sequence trace decomposition (from Brinkman et al., NAR, 2014)
2. Matlab R2014b (8.4.0.150421) routine for quantification of chromatin relaxation
3. Sanger sequence analyzer for junction analysis (from Schimmel et al., EMBO J., 2017)
4. Image J version 1.48 for microscopy image analysis
5. GraphPad Prism 8

For manuscripts utilizing custom algorithms or software that are central to the research but not yet described in published literature, software must be made available to editors and reviewers. We strongly encourage code deposition in a community repository (e.g. GitHub). See the Nature Research guidelines for submitting code & software for further information.

Data

Policy information about availability of data

All manuscripts must include a data availability statement. This statement should provide the following information, where applicable:

- Accession codes, unique identifiers, or web links for publicly available datasets
- A list of figures that have associated raw data
- A description of any restrictions on data availability

All data generated or analyzed during this study are included in this published article (and its Supplementary information files and Source data file). Additionally, the RNA-Seq data presented in Supplementary Data 2 have been deposited to the SRA database (https://www.ncbi.nlm.nih.gov/sra) with the following accession number: PRJNA547697. The mass spectrometry data presented in Supplementary Data 3 have been deposited to the ProteomeXchange Consortium via the PRIDE partner repository (https://www.ebi.ac.uk/pride/archive/login) with the data set identifier PXD014339.

Field-specific reporting

Please select the one below that is the best fit for your research. If you are not sure, read the appropriate sections before making your selection.

☒ Life sciences ☐ Behavioural & social sciences ☐ Ecological, evolutionary & environmental sciences

For a reference copy of the document with all sections, see nature.com/documents/nr-reporting-summary-flat.pdf

Life sciences study design

All studies must disclose on these points even when the disclosure is negative.

Sample size	1. Microscopy experiments aimed to acquire at least 100 cells per condition. In some cases, however, when fewer cells were acquired, the precise amount of analyzed cells is mentioned in the Figure legends. Analysis of >100 cells was sufficient to obtain normal distribution of the data and reliable mean.<br>2. Reporter assays were performed with acquisition of at least 150.000 cells per condition to obtain a clear GFP-positive cell population which accounted for ~2-5% of the total.<br>3. DSB repair junction analysis was done with ~100 sequences per condition which was sufficient to obtain enough wild type and mutated sequences for the normal distribution of the data.<br>4. Survival assays were performed with re-seeding cells in triplicate per condition to average out the variation between technical replicates.
Data exclusions	No data was excluded.
Replication	Experiments were performed at least in duplicate, but mostly in triplicate or more to asses the reproducibility. All attempts at reproduction were successful. Standard errors included in the graphs indicate the variation between replicates of each experiment.
Randomization	Experiments were performed with cell lines. For each experiments, cells were randomly seeded for different treatments.
Blinding	Blinding has not been applied, also because it is not feasible for many of the approaches used in our manuscript, including IPs, western blot analysis and siRNA/inhibitor treatments.

Reporting for specific materials, systems and methods

We require information from authors about some types of materials, experimental systems and methods used in many studies. Here, indicate whether each material, system or method listed is relevant to your study. If you are not sure if a list item applies to your research, read the appropriate section before selecting a response.

Materials & experimental systems

n/a	Involved in the study
☐	☒ Antibodies
☐	☒ Eukaryotic cell lines
☒	☐ Palaeontology and archaeology
☒	☐ Animals and other organisms
☒	☐ Human research participants
☒	☐ Clinical data
☒	☐ Dual use research of concern

Methods

n/a	Involved in the study
☒	☐ ChIP-seq
☐	☒ Flow cytometry
☒	☐ MRI-based neuroimaging

Antibodies

Antibodies used	1. Rabbit anti-53BP1 from Novus Biologicals (NB100-304)<br>2. Mouse anti-53BP1 from BD Biosciences (612522)

3. Rabbit anti-ATM from Cell Signaling (clone D2E2)
4. Mouse anti-pATM on Ser1981 from Cell Signaling (4526)
5. Mouse anti-BRCA1 from Santa Cruz (sc-6954)
6. Mouse anti-BRG1 from Santa Cruz (clone G7, sc-17796)
7. Rabbit anti-CHD7 from Bethyl Laboratories (A301-223A)
8. Rabbit anti-CHD7 from Novus Biologicals (NBP1-77393)
9. Rabbit anti-CHD4 from Active Motif (39289)
10. Mouse anti-CHK1 from Santa Cruz (clone G-4, sc-8408)
11. Rabbit anti-pCHK1 on Ser345 from Cell Signaling (clone 133D3)
12. Mouse anti-DNA-PKcs from Abcam (clone 18-2)
13. Rabbit anti-pDNA-PKcs on Ser2056 from Abcam (ab18192)
14. Mouse anti-FK2 from Enzo Lifesciences
15. Rabbit anti-Geminin from Proteintech (10802-1-AP)
16. Mouse anti-GFP from Roche (11814460001)
17. Rabbit anti-H4panAc from Millipore (06-866)
18. Rabbit anti-HDAC1 from Abcam (ab7028)
19. Rabbit anti-HDAC1 from Imgenex (IMG337)
20. Rabbit anti-HDAC2 from Santa Cruz (sc-7899)
21. Rabbit anti-Ku80 from Santa Cruz (H-300, sc-9034)
22. Rabbit anti-LIG3 from Abcam (ab96576)
24. Rabbit anti-LIG4 from Abcam (ab193353)
25. Rabbit anti-MDC1 from Abcam (ab11171-50)
26. Mouse anti-p53 from Santa Cruz (clone DO-1)
27. Rabbit anti-PAR from Enzo Lifesciences
28. Rabbit anti-PARP1 from Cell Signaling (9542)
29. Mouse anti-PCNA from Santa Cruz (PC10, sc-56)
30. Rabbit anti-RAD51 from Santa Cruz (sc-8349)
31. Mouse anti-RAD51 from GeneTex (clone 14B4)
32. Mouse anti-RNF8 from Santa Cruz (B-2)
33. Rabbit anti-RNF168 from Millipore (ABE367)
34. Mouse anti-RPA32 from Abcam (ab2175)
35. Rabbit anti-pRPA32 (S4/S8) from Bethyl Laboratories (A300-245A)
36. Mouse anti α-Tubulin from Sigma (cloneDM1A, T6199)
37. Rabbit anti-XRCC4 received as a gift from D. van Gent from Erasmus University Medical Center in Rotterdam
38. Mouse anti-XRCC4 from SAB (40455)
39. Mouse anti-gH2AX from Millipore (clone JBW301, 05-636)
40. Goat anti-rabbit CF680 from Biotium (VWR 89138-520)
41. Goat anti-mouse CF770 from Biotium (VWR 89138-532)

Validation

Commercially available antibodies were validated by the supplier.

The following antibodies were additionally validated by us in knockdown / knockout cells: Mouse anti-53BP1 from BD Biosciences (612522), Mouse anti-BRG1 from Santa Cruz (clone G7, sc-17796), Rabbit anti-CHD7 from Bethyl Laboratories (A301-223A), Rabbit anti-CHD4 from Active Motif (39289), Rabbit anti-HDAC1 from Imgenex (IMG337), Rabbit anti-HDAC2 from Santa Cruz (sc-7899), Rabbit anti-Ku80 from Santa Cruz (H-300, sc-9034), Rabbit anti-LIG3 from Abcam (ab96576), Mouse anti-XRCC4 from SAB (40455), Mouse anti-p53 from Santa Cruz (clone DO-1).

The following antibodies were validated in co-IP experiments: Mouse anti-GFP from Roche (11814460001), Rabbit anti-HDAC1 from Imgenex (IMG337), Rabbit anti-HDAC2 from Santa Cruz (sc-7899), Rabbit anti-CHD7 from Bethyl Laboratories (A301-223A).

The following antibodies were validated by western blot analysis to detect damage-induced phosphorylation: Rabbit anti-ATM from Cell Signaling (clone D2E2), Mouse anti-pATM on Ser1981 from Cell Signaling (4526), Mouse anti-CHK1 from Santa Cruz (clone G-4, sc-8408), Rabbit anti-pCHK1 on Ser345 from Cell Signaling (clone 133D3), Mouse anti-DNA-PKcs from Abcam (clone 18-2), Rabbit anti-pDNA-PKcs on Ser2056 from Abcam (ab18192), Mouse anti-RPA32 from Abcam (ab2175), Rabbit anti-pRPA32 (S4/S8) from Bethyl Laboratories (A300-245A).

The following antibody is a commonly used loading control: Mouse anti α-Tubulin from Sigma (cloneDM1A, T6199).

The following antibodies were validated in IR-induced foci and laser micro-irradiation microscopy experiments: Rabbit anti-53BP1 from Novus Biologicals (NB100-304), Mouse anti-BRCA1 from Santa Cruz (sc-6954), Mouse anti-FK2 from Enzo Lifesciences, Rabbit anti-Geminin from Proteintech (10802-1-AP), Rabbit anti-H4panAc from Millipore (06-866), Rabbit anti-PAR from Enzo Lifesciences, Rabbit anti-XRCC4 received as a gift from D. van Gent from Erasmus University Medical Center in Rotterdam, Mouse anti-gH2AX from Millipore (clone JBW301, 05-636), Rabbit anti-CHD7 from Novus Biologicals (NBP1-77393), Rabbit anti-HDAC1 from Abcam (ab7028).

Eukaryotic cell lines

Policy information about cell lines

Cell line source(s)

Human HEK293T, VH10-SV40, RPE1-hTERT, HeLa, U2OS and SV40 T-transformed GM639 human fibroblasts were used in this study.
Several cell lines were previously published:
1. RPE1-hTERT cells expressing endogenous GFP-Ku70 were a gift from Steve Jackson (Britton et al., 2013)
2. U2OS cells with stably integrated EJ5-GFP, EJ2-GFP or DR-GFP reporters were a gift from Jeremy Stark and Maria Jasin (Bennardo et al., 2008; Pierce et al., 1999)
3. U2OS cells stably expressing cell cycle markers mKO-Cdt1 and mCherry-geminin were previously generated (Luijsterburg et

al., 2016)
4. SV40 T-transformed GM639 human fibroblasts with a stably integrated GC92 reporter were a gift from Bernard Lopez (Taty-Taty et al., 2016)
5. U2OS-2B2 cells carrying a genomically integrated LacO array for use in the fluorescence three-hybrid assay were generated previously (Czarna et al., 2013)
6. U2OS cells stably expressing H2B-PAGFP were described previously (Smith et al., 2018)
7. U2OS 2-6-3 cells stably expressing ER-mCherry-LacR-FokI-DD were a gift from Roger Greenberg (Tang et al., 2013)
8. U2OS cells expressing a doxycycline-inducible shRNA against RNF168 were a kind gift from Jiri Lukas (Doil et al., 2009; Gudjonsson et al., 2012)
The remaining cell lines were purchased from ATCC.

Authentication	Cell lines were authenticated using Short Tandem Repeat (STR) analysis by ATCC services (100% match).
Mycoplasma contamination	All cell lines were routinely and regularly tested for mycoplasma and used only when non-contaminated.
Commonly misidentified lines (See ICLAC register)	No commonly misidentified cell lines were used in this study.

Flow Cytometry

Plots

Confirm that:

☒ The axis labels state the marker and fluorochrome used (e.g. CD4-FITC).

☒ The axis scales are clearly visible. Include numbers along axes only for bottom left plot of group (a 'group' is an analysis of identical markers).

☐ All plots are contour plots with outliers or pseudocolor plots.

☒ A numerical value for number of cells or percentage (with statistics) is provided.

Methodology

Sample preparation	2 days after I-Sce-I transfection cells carrying a DSB reporter were trypsinized and diluted in PBS supplemented with 2% FBS
Instrument	BD LSRII flow cytometer (BD Bioscience)
Software	FACSDiva software version 5.0.3. (BD Bioscience)
Cell population abundance	At least 150.000 cells/events were acquired for each condition
Gating strategy	To analyze GFP and mCherry positive U2OS cells carrying the DSB reporters (EJ5-GFP, EJ2-GFP, DR-GFP), three initial gates were set in the following sequential plots: 1) SSC-A scatter (Y-axis) set out against FSC-A scatter (X-axis), allowing us to set gate P1 for living cells, 2) FSC-H scatter (Y-axis) set out against FSC-W scatter (X-axis), allowing us to set gate P2, and 3) SSC-H scatter (Y-axis) set out against SSC-W scatter (X-axis), allowing us to set gate P3. Gates P2 and P3 allowed us to exclude doublets. In a fourth plot (GFP-A on Y-axis and mCherry-A on X-axis), mCherry positive cells were scored using a gating based on mCherry negative control cells (gate P4). Subsequent gate P5 was positioned within Chery-positve population (within gate P4) to gate for GFP positive cells.<br><br>To analyze GFP positive U2OS cells transfected with pEGFP construct in random plamsmid integration assays, three initial gates were set in the following sequential plots: 1) SSC-A scatter (Y-axis) set out against FSC-A scatter (X-axis), allowing us to set gate P1 for living cells, 2) FSC-H scatter (Y-axis) set out against FSC-W scatter (X-axis), allowing us to set gate P2, and 3) SSC-H scatter (Y-axis) set out against SSC-W scatter (X-axis), allowing us to set gate P3. Gates P2 and P3 allowed us to exclude doublets. In a fourth plot (SSC-A on Y-axis and GFP-A on X-axis), GFP positive cells were scored using a gating based on GFP negative control cells (gate P4).

☒ Tick this box to confirm that a figure exemplifying the gating strategy is provided in the Supplementary Information.
